# Polymerase pausing induced by sequence-specific RNA-binding protein drives heterochromatin assembly

**DOI:** 10.1101/gad.310136.117

**Published:** 2018-07-01

**Authors:** Jahan-Yar Parsa, Selim Boudoukha, Jordan Burke, Christina Homer, Hiten D. Madhani

**Affiliations:** 1 Department of Biochemistry and Biophysics, University of California at San Francisco, San Francisco, California 94158, USA;; 2 Chan-Zuckerberg Biohub, San Francisco, California 94158, USA

**Keywords:** heterochromatin, Seb1, polymerase pausing, centromere

## Abstract

In *Schizosaccharomyces pombe*, transcripts derived from the pericentromeric *dg* and *dh* repeats promote heterochromatin formation via RNAi as well as an RNAi-independent mechanism involving the RNA polymerase II (RNAPII)-associated RNA-binding protein Seb1 and RNA processing activities. We show that Seb1 promotes long-lived RNAPII pauses at pericentromeric repeat regions and that their presence correlates with the heterochromatin-triggering activities of the corresponding *dg* and *dh* DNA fragments. Globally increasing RNAPII stalling by other means induces the formation of novel large ectopic heterochromatin domains. Such ectopic heterochromatin occurs even in cells lacking RNAi. These results uncover Seb1-mediated polymerase stalling as a signal necessary for heterochromatin nucleation.

## Abstract (precis)

In this study, Parsa et al. investigated the mechanisms underlying RNAi-independent heterochromatin assembly by the CTD–RRM protein Seb1 in *S. pombe*. They show that Seb1 promotes long-lived RNAPII pauses at pericentromeric repeat regions and that their presence correlates with the heterochromatin-triggering activities of the corresponding *dg* and *dh* DNA fragments, providing new insight into Seb1-mediated polymerase stalling as a signal necessary for heterochromatin nucleation.

Packaging of pericentromeric DNA into heterochromatin is crucial for genome stability, development, and health, yet its endogenous triggers remain poorly understood (Allshire and Madhani 2018). A defining feature of pericentromeric heterochromatin is histone H3 Lys9 methylation (H3K9me) (Rea et al. 2000; Bannister et al. 2001; Lachner et al. 2001). Investigations of the fission yeast *Schizosaccharomyces pomb*e have played a major role in our understanding of this type of repressive chromatin. In *S. pombe*, transcripts derived from the pericentromeric *dg* and *dh* repeats during S phase (Volpe et al. 2002; Chen et al. 2008; Kloc and Martienssen 2008) promote heterochromatin formation through two parallel pathways. The first is an RNAi-dependent mechanism involving recruitment of the Clr4 H3K9 methyltransferase complex (CLR-C) via the Argonaute-containing RNA-induced transcriptional silencing (RITS) complex (Motamedi et al. 2004; Noma et al. 2004; Verdel et al. 2004; Sugiyama et al. 2005; Buhler et al. 2006; Bayne et al. 2010).

A second, less-characterized pathway does not require RNAi factors. Components that impact this mechanism include the RNA polymerase II (RNAPII)-associated RNA-binding protein Seb1, the repressor/remodeler complex SHREC, the nuclear 5′–3′ exonuclease Dhp1 (Rat1/Xrn2 in *Saccharomyces cerevisiae*), the nuclear 3′–5′ exonuclease Rrp6, and the RNA export factor Mlo3/Yra1 (Sadaie et al. 2004; Sugiyama et al. 2007; Reyes-Turcu et al. 2011; Marina et al. 2013; Chalamcharla et al. 2015; Lemay et al. 2016; Tucker et al. 2016; Wittmann et al. 2017). Seb1, Dhp1, and SHREC promote RNAi-independent pericentromeric heterochromatin assembly at pericentromeric regions (Marina et al. 2013; Chalamcharla et al. 2015; Tucker et al. 2016), while Rrp6 and Mlo3 inhibit it (Reyes-Turcu et al. 2011). Other fungi, such as *Neurospora crassa* and *Cryptococcus neoformans*, as well as somatic mammalian cells do not require RNAi for heterochromatin assembly (Freitag et al. 2004; Wang et al. 2010; Chan and Wong 2012); thus, poorly understood RNAi-independent mechanisms are important to investigate. In this study, we show that Seb1, whose role in heterochromatin we established in prior work (Marina et al. 2013), displays extensive binding to *dg* and *dh* repeat RNAs and promotes long-lived pausing by RNAPII. We show that inducing pausing by other means can trigger the assembly of ectopic heterochromatin domains independently of RNAi. The cleavage polyadenylation (CPA) machinery antagonizes silencing, suggesting a possible mechanism by which heterochromatin is limited at mRNA-coding genes despite the presence of termination-associated pauses. These findings establish a role for Seb1-dependent RNAPII pausing in promoting the formation of repressive chromatin structures.

## Results

### Nascent elongating transcript sequencing (NET-seq) reveals a role for Seb1 in the pausing of RNAPII

**Figure 1.**
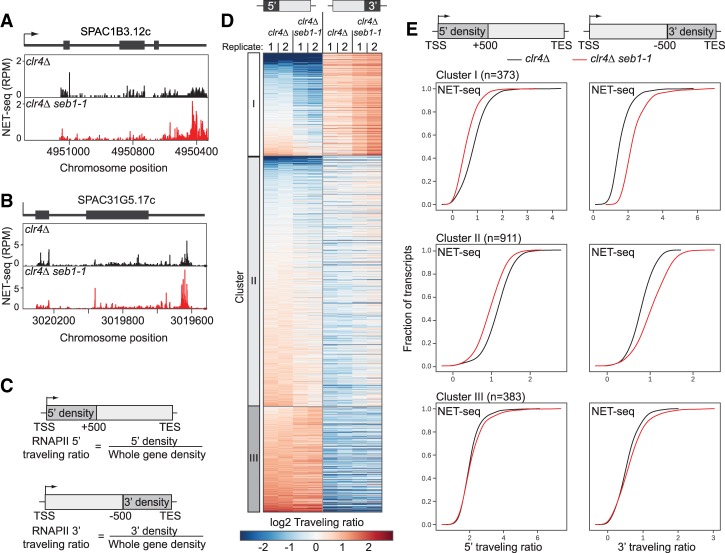
Seb1 controls polymerase progression. (*A*,*B*) NET-seq signatures for *clr4Δ* (black) and *clr4Δ seb1-1* (red) strains for two genes (SPAC1B3.12c and SPAC31G5.17c). (*C*) Traveling ratios at the 5′ and 3′ regions of genes. (*D*) *K*-means clustering of NET-seq 5′ and 3′ traveling ratios. Cluster I, *n* = 373; cluster II, *n* = 911; cluster III, *n* = 383. NET-seq replicates are represented for each *clr4Δ* and *clr4Δ seb1-1*. (*E*) Cumulative distribution function (CDF) plots of 5′ (*left* column) and 3′ (*right* column) traveling ratios for each cluster from *D*, comparing *clr4Δ* (black) with *clr4Δ seb1-1* (red) strains in each plot. KS tests were conducted for *P*-values (Supplemental Fig. S1).

To understand how Seb1 interfaces with the transcription of *dg* and *dh* repeats to promote heterochromatin, we used a previously identified viable heterochromatin-defective allele, *seb1-1* (Marina et al. 2013). When combined with mutants in the RNAi machinery, *seb1-1* eliminates pericentromeric heterochromatin, while the corresponding single mutants decrease H3K9me, indicative of partially redundant pathways (Marina et al. 2013). We examined transcription of heterochromatin at single-nucleotide resolution and tested the impact of the *seb1-1* allele using NET-seq (Churchman and Weissman 2011). To analyze the intrinsic transcriptional properties of heterochromatic sequences prior to the establishment of heterochromatin assembly, we used the *clr4Δ* mutant, which lacks H3K9me and displays full derepression of most silenced chromatin regions. We compared this strain with a *clr4Δ seb1-1* double mutant to assess the impact of *seb1-1*. We first examined the effect of *seb1-1* on transcription of nonheterochromatic regions (Fig. 1). Initial inspection revealed numerous genes with a decreased peak density at 5′ regions in the double mutant with increased peak density upstream of annotated CPA sites (often called transcription end sites [TESs]) (see Fig. 1A,B for examples). To analyze these trends genome-wide, travelling ratios were computed on replicate data to assess relative polymerase progression for the 500-base-pair (bp) segment downstream from the transcription start site (TSS; 5′ traveling ratio) and the 500-bp segment upstream of the annotated TES (3′ traveling ratio) (Fig. 1C; see the Materials and Methods). A lower travelling ratio in mutant versus wild type implies lower pausing over the region examined in the mutant and vice versa for higher ratios. Iterative *K*-means clustering revealed three groups (Fig. 1D), two of which (representing 77% of genes in our data set) are significantly impacted by the *seb1-1* allele (Fig. 1D, clusters I and II; Supplemental Fig. S1). The *seb1-1* mutation causes a reduced median 5′ traveling ratio and an increased median 3′ traveling ratio for both clusters (Fig. 1D [clusters I and II], E [top and middle panels]), while no significant changes were observed for the third cluster (Fig. 1E, cluster III, bottom panel; see Supplemental Fig. S1 for *P*-values). These data indicate that the *seb1-1* allele leads to decreased RNAPII pausing at gene 5′ ends with an associated increased 3′ signal; the latter may be due to polymerase release from upstream pauses.

### A direct role for Seb1 in RNAPII pausing and heterochromatin assembly in the pericentromeric *dh* and *dg* repeats

**Figure 2.**
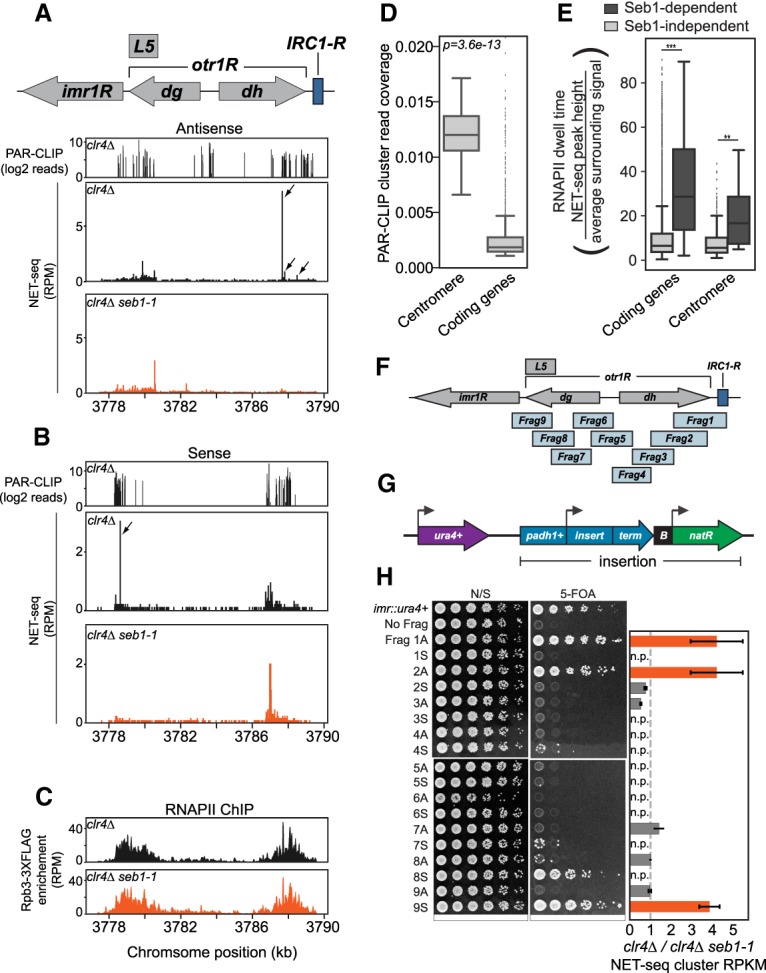
Seb1 directly binds to pericentromeric transcripts and induces RNAPII pausing in silencing-promoting segments. (*A*,*B*) Seb1 PAR-CLIP read clusters (log_2_ reads) and NET-seq peaks (reads per million mapped reads [RPM]) aligned to the right arm of centromere 1 in *clr4Δ* (PAR-CLIP) or comparing *clr4Δ* (black) and *clr4Δ seb1-1* (red) strains (NET-seq). (*A*) Read clusters/peaks aligning to the antisense transcript. (*B*) Read clusters/peaks aligning to the sense transcript. Arrows indicate locations of Seb1-dependent NET-seq peak clusters identified computationally (see the Materials and Methods). (*C*) ChIP-seq (chromatin immunoprecipitation [ChIP] combined with high-throughput sequencing) data of Rpb3-3xFlag for the right arm of centromere 1, comparing RNAPII enrichment in *clr4Δ* (black) and *clr4Δ seb1-1* (red). (*D*) Seb1 PAR-CLIP read cluster coverage at the centromeres compared with coding genes. (*E*) RNAPII dwell time analysis for centromeres and coding genes, comparing Seb1-dependent (dark gray) and Seb1-independent (light gray) pauses. (**) *P* = 0.0068; (***) *P* = 3.6 × 10^−59^. (*F*) Illustration of the right arm of centromere 1 and nine overlapping fragments analyzed. (*G*) Illustration of the reporter construct used to determine the silencing capacity of centromere fragments shown in *F*. (*padh1*^+^) *adh1*^+^ promoter; (term) a bidirectional terminator; (B) B-boxes boundary element; (*natR*) nourseothricin-resistance gene. The construct was placed downstream from *ura4*^+^. (*H*) Silencing assays for each fragment in antisense (A) or sense (S) orientations (spotting assay). Cells were plated on nonselective YS medium (N/S) and YS medium supplemented with 5-FOA (5-FOA). Controls for plating were a strain encoding a single functional *ura4*^+^ gene placed in the innermost repeats of centromere 1 (*imr::ura4*^+^) and a strain with the construct in *G* containing no fragment (No Frag). Ratios of *clr4Δ*/*clr4Δ seb1-1* for NET-seq clusters in each fragment in antisense and sense transcription units (bar graph). Value of 1 represents no change in NET-seq signal (dotted line), and a value of >1 represents NET-seq clusters in *clr4Δ* that are reduced in *clr4Δ seb1-1*. Silencing-competent fragments are orange, and silencing-deficient fragments are dark gray. (n.p.) No peaks.

Our prior RIP-qPCR (RNA immunoprecipitation [RIP] combined with quantitative PCR [qPCR]) analysis indicates that Seb1 functions directly in heterochromatin assembly by binding pericentromeric *dg* and *dh* repeat transcripts (Marina et al. 2013). Furthermore, comparison of the transcriptomes of wild-type and *seb1-1* using RNA sequencing (RNA-seq) revealed no significant changes [*P* < 0.01 and |log_2_(fold change)| > 1] in the transcript levels of known silencing factors (Supplemental Fig. S2; Supplemental Table S1). To assess direct interactions of Seb1 with pericentromeric RNA at single-nucleotide resolution and across the entirety of the *dg* and *dh* regions, we conducted PAR-CLIP (photoactivatable ribonucleoside-enhanced cross-linking and immunoprecipitation) in replicate on a *clr4Δ* strain. Computational analysis identified statistically significant Seb1 PAR-CLIP read clusters (Corcoran et al. 2011) and confirmed direct binding of Seb1 to pericentromeric transcripts (Fig. 2A,B, top panels; Supplemental Fig. S3A–C) via a motif described previously by others for nonheterochromatic sites bound by Seb1 (Lemay et al. 2016; Wittmann et al. 2017): UGUA (DREME motif analysis; *P* = 2.9 × 10^−9^; *E* = 7.4 × 10^−7^) (Bailey 2011). Our analysis of Seb1 PAR-CLIP read clusters for coding genes recapitulates published work and is not discussed further here (Lemay et al. 2016; Wittmann et al. 2017).

To compare the binding of Seb1 across transcript classes, we computed the fraction of RNA covered by Seb1 PAR-CLIP read clusters. We observed an ∼12-fold higher PAR-CLIP cluster coverage for pericentromeric repeat intervals than for coding gene intervals (Fig. 2D; Supplemental Fig. S4A). Noncoding RNAs (ncRNAs) display the highest coverage at a mean level ∼100-fold higher than that of coding genes (Supplemental Fig. S4B).

We next examined the NET-seq profiles of pericentromeric heterochromatin sequences of *clr4Δ* and *clr4Δ seb1-1* strains (replicate experiments were conducted). Pericentromeric regions harbor detectable NET-seq signal in *clr4Δ* cells despite a low level of polymerase engagement at any single nucleotide (Fig. 2A,B, middle panels). The signal overlaps with regions of high Seb1 PAR-CLIP cluster coverage (Fig. 2A,B, top panels). Notably, a handful of discrete peaks indicative of pausing were observed, and the largest were lost in the *seb1-1* mutant for both antisense and sense signals (Fig. 2A,B, bottom panels; Supplemental Table S2). RNAPII densities at the pericentromeric regions are comparable between *clr4Δ* and *clr4Δ seb1-1* strains as assessed by ChIP-seq (chromatin immunoprecipitation [ChIP] combined with high-throughput sequencing) analysis of Rpb3-3xFlag (Fig. 2C; Supplemental Fig. S4C), indicating that decreases in NET-seq peak intensities caused by the *seb1-1* mutation are not trivially explained by loss of RNAPII recruitment (note that rare pauses may not impact overall RNAPII density as measured by ChIP-seq). Notably, calculation of polymerase dwell times (Larson et al. 2014) at centromeres and across the genome and genotypes revealed that Seb1-dependent pauses are significantly longer-lived on average than Seb1-independent pauses (Fig. 2E; Supplemental Fig. S4D). These data reveal detectable Seb1-dependent RNAPII pauses in pericentromeric sequences.

Previous studies identified two segments of pericentromeric DNA that can trigger heterochromatin: *L5* (Partridge et al. 2002; Volpe et al. 2003) and fragment 1 (*Frag1*) (Marina et al. 2013), respectively. *Frag1* defined a segment that requires both RNAi and Seb1 for its activity (Marina et al. 2013). To compare the activity of pericentromeric fragments with their transcription properties described above, we extended this analysis using a system that we used previously (Marina et al. 2013). The *cen1R* region was divided into nine overlapping fragments (Fig. 2F; Supplemental Table S3). Each fragment was placed downstream from an *adh1*^+^ promoter (*padh1*^+^) in either forward or reverse orientation and upstream of a transcription terminator. This insert was then placed downstream from *ura4*^+^ (Fig. 2G). Silencing of *ura4*^+^ was determined using YS-FOA plates, which select for *ura4*^+^ repression. The insert of *Frag1* displays silencing activity; this construct was used previously to isolate the *seb1-1* mutant and was shown to require the *padh1*^+^ promoter for silencing activity (Marina et al. 2013). Three additional fragments exhibit strong silencing activity, and each is functional in only one orientation (*Frag2A*, *Frag8S*, and *Frag9S*) (Fig. 2H). Thus, these pericentromeric regions harbor a transcription-dependent orientation-specific signal capable of triggering silencing. To examine the relationship of these regions to those that display detectable Seb1-dependent pauses, we identified clusters of NET-seq peaks (see the Materials and Methods) and computed the total read density of these clusters within each fragment. A comparison of *clr4Δ* to *clr4Δ seb1-1* strains revealed significant correlation (χ^2^ = 12.6, *P* < 0.001) (Fig. 2H, *Frag1A*, *Frag2A*, and *Frag9S*; Supplemental Fig. S5A–C). The exception, *Frag8S,* displays silencing activity but no detectable Seb1-dependent NET-seq peak clusters (although it does display Seb1-dependent NET-seq signal) (Fig. 2H; Supplemental Fig. S5D). The heterochromatin assembly activity of this fragment may be pause-independent, or the relevant RNAPII pauses may be below the sensitivity of NET-seq. Introduction of the *seb1-1* allele into these reporter strains (*Frag2A*, *Frag8S*, and *Frag9S*) by homologous recombination leads to reduced silencing of *ura4*^+^ (Supplemental Fig. S5E). The residual activity is likely due to the parallel RNAi pathway (Marina et al. 2013). These data indicate that Seb1 directly recognizes *dg* and *dh* RNAs, induces detectable pausing in centromere fragments, and promotes the silencing activity directed by these sequences.

### Global enhancement of RNAPII pausing can trigger ectopic heterochromatin assembly

**Figure 3.**
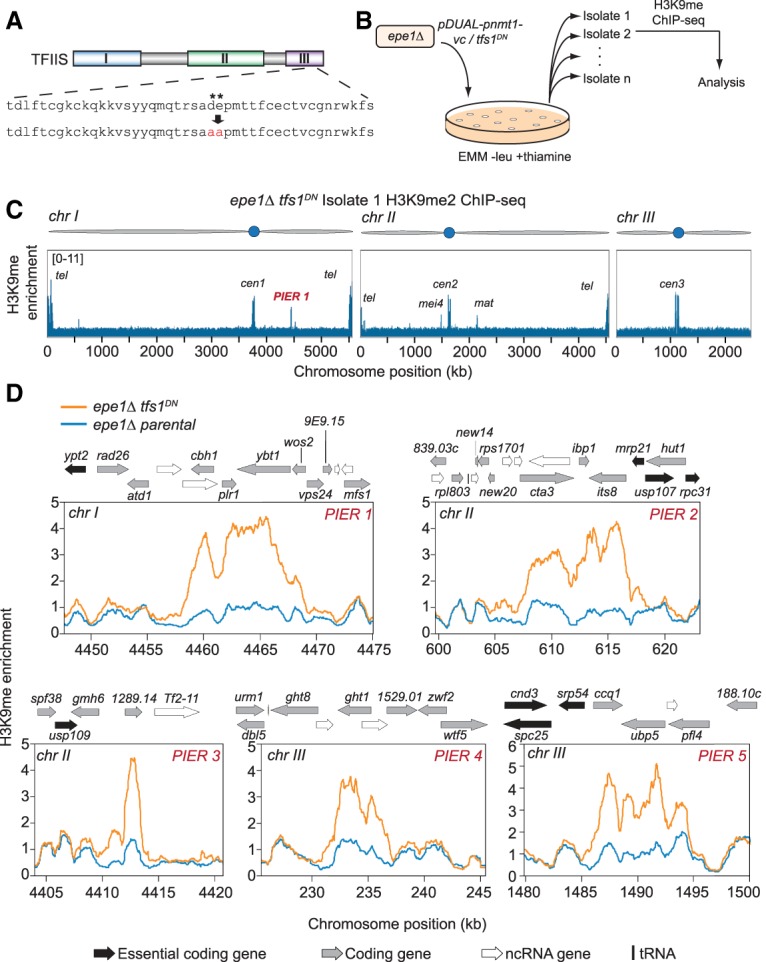
TFIIS^DN^ induces ectopic heterochromatin formation. (*A*) Representation of the domains present in TFIIS. Two acidic residues in domain III (denoted by asterisks) were mutated to alanine residues to produce TFIIS^DN^. (*B*) Pipeline for isolating TFIIS^DN^-expressing cells and analysis of genome-wide H3K9me2. *S. pombe epe1Δ* strains were transformed with *tfs1*^*DN*^ controlled by the *nmt1*^+^ thiamine-repressible promoter (*pDUAL-pnmt1^+^-tfs1*^*DN*^) or a vector control (*pDUAL-pnmt1^+^-vc*) and selected on Edinburgh minimal medium (EMM) −leu +thiamine plates. Thirteen isolates for each *epe1Δ tfs1*^*DN*^ and 15 isolates for *epe1Δ* vector control strains were collected. ChIP-seq for H3K9me2 was conducted and analyzed for each isolate as well as a parental strain for each set of isolates. (*C*) Genome-wide representation of H3K9me2 ChIP-seq enrichment for *epe1Δ tfs1*^*DN*^ isolate 1. (PIER) Pause-induced ectopic heterochromatic region. (*D*) Genome browser images of PIERs 1–5 that were observed from *epe1Δ tfs1*^*DN*^ isolates. Each plot contains the H3K9me2 enrichment of the parental *epe1Δ* strain (blue) and the *epe1Δ tfs1*^*DN*^ strain (orange). Genome features are displayed *above* each browser image. (Black arrow) Essential coding gene; (gray arrow) coding gene; (white arrow) ncRNA; (black bar) tRNA.

The shared defect of the viable *seb1-1* allele in both heterochromatin assembly and RNAPII pausing suggests that pausing signals the assembly of heterochromatin. However, given that Seb1 may have other activities impacted by *seb1-1*, we sought an orthogonal test of the role of pausing. Thus, we pursued an alternative strategy of testing whether increasing RNAPII pausing per se could be sufficient to trigger heterochromatin assembly and, if so, whether such an activity required RNAi. We exploited the conserved elongation factor TFIIS, which binds paused RNAPII complexes and stimulates RNA hydrolysis by RNAPII, enabling restart (Mayer et al. 2017). It is thought that all genes are subject to this type of rescue mechanism, as pausing is a ubiquitous feature of transcription. Two conserved acid residues in domain III of TFIIS are required for catalysis (Jeon et al. 1994). Mutation of these residues to alanine prevents the cleavage of the RNA by RNAPII (Jeon et al. 1994), ultimately resulting in polymerase trapped in a lethal paused/backtracked state (Sigurdsson et al. 2010; Imashimizu et al. 2013). We introduced the corresponding D274A and E275A mutations in the TFIIS gene *tfs1*^+^, creating a dominant-negative allele, *tfs1*^*DN*^ (Fig. 3A; Lemay et al. 2014). To overcome lethality of this allele (Sigurdsson et al. 2010; Imashimizu et al. 2013), we placed it under control of an *nmt1*^+^ thiamine-repressible promoter and inserted it at the *leu1*^+^ locus (Matsuyama et al. 2004), enabling concerted expression of *tfs1*^+^ and *tfs1*^*DN*^. We conducted replicate NET-seq analysis on *clr4Δ* and *clr4Δ tfs1*^DN^ cells under inducing conditions. Genome-wide analysis of 5′ and 3′ traveling ratios indicated TFIIS^DN^-dependent increases in RNAPII pausing at gene 5′ ends and a more modest effect at 3′ ends (Supplemental Fig. S6A,B).

To test whether stabilizing endogenous RNAPII pauses in this manner triggers de novo ectopic heterochromatin, we performed H3K9me2 ChIP-seq analysis on *tfs1*^*DN*^ strains; however, we observed no effects in this background (data not shown). Heterochromatin components are limiting and antagonized by anti-silencing factors, particularly Epe1, which actively removes the H3K9me mark (Zofall and Grewal 2006; Aygun et al. 2013; Audergon et al. 2015). Thus, we constructed *epe1Δ tfs1*^*DN*^ mutant strains or *epe1Δ* strains carrying an integrated vector-only control (*epe1Δ-vc*), collected multiple strain isolates for each, and performed H3K9me2 ChIP-seq on all isolates (Fig. 3B). Because high-level TFIIS^DN^ expression is lethal in *epe1Δ* cells (Supplemental Fig. S6C, middle panel), experiments were performed in the presence of thiamine, enabling viability (Supplemental Fig. S6C, right panel). Consistent with a slight fitness defect under these conditions (Supplemental Fig. S6C, right panel), RNA-seq analysis revealed that 13% of the transcript pool from the *tfs1* genes arise from the *tfs1*^*DN*^ allele, and 87% arise from the wild-type allele when cells are grown in thiamine (Supplemental Fig. S6D), indicating leaky repression. The ChIP-seq data obtained from strain isolates were examined for H3K9me peaks (see the Materials and Methods). We filtered the results for well-established heterochromatic recruitment sites (including HOODs, islands, meiotic genes, Epe1-bound genes, etc.) (see the Materials and Methods), as these genomic regions have an intrinsic propensity (e.g., via RNAi or the RNA elimination machinery) to nucleate H3K9me (Zofall et al. 2012; Yamanaka et al. 2013; Wang et al. 2015).

Remarkably, of 13 isolates derived from *epe1Δ tfs1*^*DN*^ parents analyzed by H3K9me ChIP-seq, five separate isolates harbor a distinct ectopic region of heterochromatin, which we termed the pause-induced ectopic heterochromatic region (PIER) (Fig. 3C,D; Supplemental Fig. S7). No novel ectopic heterochromatic loci were observed by whole-genome H3K9me2 ChIP-seq in the 15 *epe1Δ-vc* strains (χ^2^ = 7.02, *P* = 0.0082). PIERs range in size from ∼3 to ∼15 kb, and each PIER was unique. Three PIERs are bounded by an essential gene on at least one side of the locus, suggesting that selection likely prevents observing PIERs that assemble over essential genes (PIER2, PIER3, and PIER5) (Fig. 3D); this implies that our approach may underestimate the propensity of PIER formation. H3K9me enrichment at known heterochromatin nucleation sites is unrelated to *tfs1* genotype and is summarized for all strains in Supplemental Figure S8. These results indicate that TFIIS^DN^-induced RNAPII pausing can be sufficient to nucleate heterochromatin at novel sites.

To determine whether H3K9me at PIERs leads to repression, we conducted RNA-seq analysis on *epe1Δ tfs1*^*DN*^ isolate 2 (containing PIER2) (Supplemental Fig. S9A; Supplemental Table S4). We observed a significant decrease (*P* < 0.01) in two of the three genes present in PIER2: *cta3*^+^ and *its8*^+^ (Supplemental Fig. S9A; Supplemental Table S4). TFIIS^DN^ expression does not alter the expression of known heterochromatin factors [*P* < 0.01, |log_2_(fold change)| > 1] (Supplemental Fig. S9B; Supplemental Table S4).

### PIER induction is independent of RNAi

**Figure 4.**
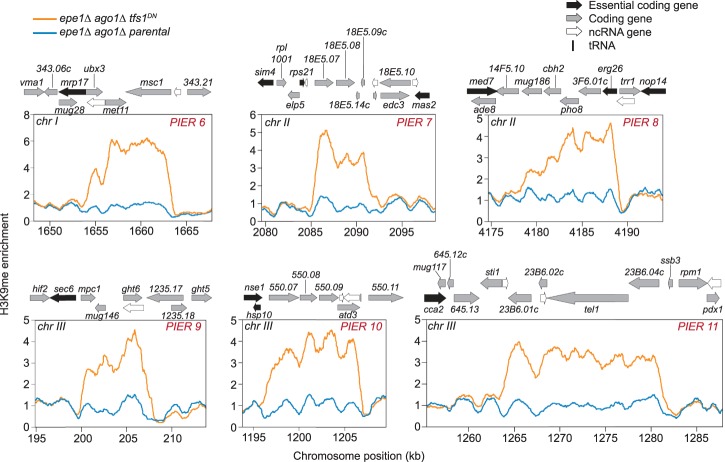
TFIIS^DN^-induced ectopic heterochromatin is RNAi-independent. Genome browser images of PIER6–11 that were observed from *epe1Δ ago1Δ tfs1*^*DN*^ isolates. *epe1Δ ago1Δ* strains were transformed with *pDUAL-pnmt1^+^-tfs1*^*DN*^, and H3K9me2 ChIP-seq was conducted. Genome-wide H3K9me2 enrichment was analyzed, and six PIERs (PIER6–11) were observed from *epe1Δ ago1Δ tfs1*^*DN*^. Each plot contains the H3K9me2 enrichment of the parental *epe1Δ ago1Δ* strain (blue) and the *epe1Δ ago1Δ tfs1*^*DN*^ strain (orange). Genome features are displayed *above* each browser image. (Black arrow) Essential coding gene; (gray arrow) coding gene; (white arrow) ncRNA; (black bar) tRNA.

Four out of five PIERs contain overlapping convergent genes that have the potential to form dsRNA (PIER1, PIER2, PIER4, and PIER5) (Fig. 3D). As dsRNA can trigger heterochromatin in *cis* via the RNAi pathway (Simmer et al. 2010), we determined whether the de novo establishment of PIERs requires RNAi. We constructed *epe1Δ ago1Δ* strains by disrupting *ago1*^+^ in the *epe1Δ* mutant. Initial ChIP-seq analysis of three isolates revealed two that display ectopic heterochromatin at the *clr4*^+^ locus and reduced levels of H3K9me at constitutive heterochromatic loci (Supplemental Figs. S10, S11B,C, top panels). Such adaptive silencing of *clr4*^+^ has been described previously in *epe1Δ mst2Δ* strains (Wang et al. 2015) and evidently can occur in strains lacking Epe1 and RNAi. Remarkably, upon integration of *tfs1*^*DN*^ into these two *epe1Δ ago1Δ* strains, H3K9me2 ChIP-seq revealed establishment of heterochromatin at constitutively silenced loci (e.g., centromeres) and its loss at the *clr4*^+^ locus (Supplemental Fig. S11B,C, isolates 1, 3, 4, and 5). This observation indicates that pericentromeric repeats harbor the ability to respond to RNAPII pausing and assemble heterochromatin independently of RNAi. Accompanying these changes, six out of the 12 *epe1Δ ago1Δ tfs1*^*DN*^ isolates subjected to whole-genome analysis acquired PIERs (Fig. 4; Supplemental Fig. S11). Five of six of these PIERs are bounded by an essential gene on at least one side of the region similar to that seen in the *epe1Δ tfs1*^*DN*^ (PIER6–10) (Fig. 4), consistent with the notion that essential genes limit our ability to observe ectopic heterochromatin. Again, each PIER was unique. Thus, PIERs can be triggered by RNAPII pausing even in cells lacking a functional RITS complex.

### The CPA machinery antagonizes heterochromatin assembly

**Figure 5.**
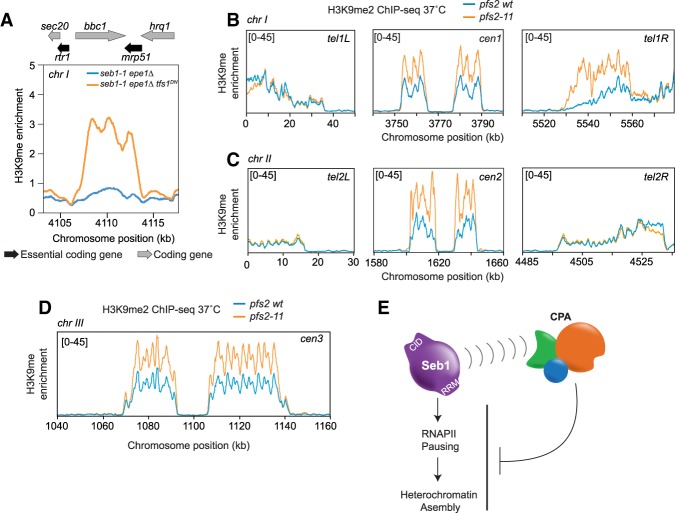
Seb1 promotes PIER formation, and the CPA machinery limits heterochromatin assembly. (*A*) Graph of the single PIER from *seb1-1 epe1Δ tfs1*^*DN*^ isolates. The plot depicts the H3K9me2 enrichment of the parental *seb1-1 epe1Δ* strain (blue) and the *seb1-1 epe1Δ tfs1*^*DN*^ strain (orange). Genome features are displayed *above* the image. (Black arrow) Essential coding gene; (gray arrow) coding gene. (*B*–*D*) Images of H3K9me enrichment at the telomeres and centromere of chromosome I (*B*), chromosome II (*C*), and the centromere III (*D*) in wild-type (blue) and *pfs2-11* (orange) mutant backgrounds after temperature shift. (*E*) Model depicting activation of heterochromatin assembly by Seb1-dependent RNAPII pausing and its repression by the CPA machinery.

To address the role of Seb1 in the formation of PIERs, we interrogated 15 independent isolates of *seb1-1 epe1Δ tfs1*^*DN*^ for ectopic heterochromatin assembly by whole-genome ChIP. A single PIER was observed (Fig. 5A)—a frequency significantly lower than that produced by *epe1Δ tfs1*^*DN*^ strains (χ^2^ = 4.18, *P* = 0.0409). Two additional regions of ectopic heterochromatin appear evident by visual inspection but were below our cutoffs for H3K9me enrichment (Supplemental Fig. S12A–C). These data suggest that while frequent PIER formation and high levels of H3K9me accumulation are promoted by Seb1, the *seb1-1* allele is permissive for some degree of H3K9me triggered by *tfs1*^*DN*^.

Our data demonstrate that Seb1 promotes RNAPII pausing at centromeres, the major site of constitutive heterochromatin assembly, but also at numerous additional sites (Fig. 1). Why, then, is heterochromatin assembly restricted to specific sites under standard growth conditions? A potential clue comes from studies that show that Seb1 promotes CPA termination and copurifies with the CPA machinery (Lemay et al. 2016; Wittmann et al. 2017). One possibility is that recruitment of the CPA by strong polyadenylation signals might suppress heterochromatin assembly, thereby neutralizing the heterochromatin-promoting effects of Seb1. To test this idea, we used a well-characterized temperature-sensitive mutant of a CPA factor, *pfs2-11*, which produces transcription readthrough past the CPA site under nonpermissive conditions (Wang et al. 2005). Strikingly, under these conditions, we observed increased levels of H3K9me at all centromeres (Fig. 5B–D; Supplemental Fig. S13) as well as increased heterochromatin assembly over a 15-kb subtelomeric region on the right arm of chromosome 1 (Fig. 5B; Supplemental Fig. S13). Notably, the increase in centeromeric H3K9me is not at the expense of its loss elsewhere, as occurs in some mutant backgrounds due to limiting amounts of silencing factors (Allshire and Madhani 2018). These data support a model in which the CPA machinery or its actions suppresses heterochromatin formation at sites of Seb1-promoted CPA (Fig. 5E). They are also consistent with the observation that deletion of sequences that include polyadenylation signal enhance heterochromatin assembly on a reporter gene triggered in *trans* by expression of an artificial hairpin (Yu et al. 2014).

## Discussion

Our results indicate that Seb1, a conserved RNAPII-associated RNA-binding protein that mediates RNAi-independent heterochromatin assembly in *S. pombe* (Marina et al. 2013), is enriched on pericentromeric ncRNA transcripts relative to coding sequences and promotes long-lived RNAPII pauses. Remarkably, pausing is sufficient to trigger ectopic heterochromatin assembly in an RNAi-independent fashion, indicating that this is a relevant activity of Seb1 in promoting heterochromatin assembly. Binding of Seb1 to euchromatic ncRNAs (e.g., small nuclear RNAs [snRNAs]) is not associated with detectable heterochromatin assembly, which may be due to high levels of transcription, which induces anti-silencing histone marks and histone turnover, both of which antagonize silencing (Allshire and Madhani 2018). At mRNA-coding genes, heterochromatin assembly suppression by the CPA machinery may also play a role in specificity (Fig. 5E). Additionally, the repetitiveness of pericentromeric sequences may also contribute to specificity by producing a threshold density of paused polymerases within a discrete genomic interval. Testing these and other possibilities will require the development of tools that enable the programming of pauses of defined length at defined sites and at defined levels of transcription. Relevant to the issue of heterochromatin assembly specificity is a recent study (Gallagher et al. 2018) that reported that the low-temperature culturing of *S. pombe* triggers H3K9me heterochromatin islands across the genome independently of RNAi. Thus, the ability of “euchromatic” sites to assemble facultative heterochromatin is evidently higher than might have been assumed.

Our data are germane to the observation that mutations in the Paf1 complex (Paf1-C), a multifunctional elongation complex that binds cooperatively to RNAPII with TFIIS (Xu et al. 2017), enables synthetic hairpin RNAs to trigger heterochromatin in *trans* and increases heterochromatin spreading in *S. pombe* (Kowalik et al. 2015; Sadeghi et al. 2015; Verrier et al. 2015). While the elongation-promoting activity of Paf1-C has been suggested to limit heterochromatin by limiting targeting of RITS to the nascent transcript (Kowalik et al. 2015), it may also act via RNAi-independent mechanisms, as we found that increased RNAPII pausing can trigger H3K9me independently of RNAi. Seb1-triggered RNAPII pausing may drive heterochromatin assembly by promoting the heterochromatic stalling of replisomes associated with CLR-C through RNAPII–replisome collisions as proposed (Li et al. 2011; Zaratiegui et al. 2011). Analogous concepts have been put forth in *S. cerevisiae*, where tight protein–DNA interactions are sufficient to trigger recruitment of the SIR complex (Dubarry et al. 2011). Consistent with this hypothesis, such transcription–replication conflicts are limited by Paf1-C (Poli et al. 2016), which inhibits heterochromatin assembly, while slowing of replisome progression enhances heterochromatin spread (Singh and Klar 2008; Li et al. 2017). It has also been proposed that the 5′ → 3′ RNA exonuclease Dhp1 (related to *S. cerevisiae* Rat1/Xrn2), which is required for RNAi-independent heterochromatin assembly, recruits the silencing machinery via a physical interaction with CLR-C (Chalamcharla et al. 2015; Tucker et al. 2016). Because RNAPII pausing enhances recruitment of Xrn2 (Wagschal et al. 2012; Contreras et al. 2013), and Seb1 copurifies with the Dhp1 (Lemay et al. 2016; Wittmann et al. 2017), Seb1-induced pausing may promote heterochromatin assembly via this mechanism as well. Our model also readily accommodates genetic observations that null mutants in *S. pombe* RNAPII elongation factors suppress the H3K9me defect of RNAi mutants (Reyes-Turcu et al. 2011; Sadeghi et al. 2015) as well as analogous observations for mutants in RNA biogenesis factors (Reyes-Turcu et al. 2011), as these factors also promote transcriptional elongation (Luna et al. 2012). Weak CPA signals promote heterochromatin assembly (Yu et al. 2014), which is predicted to result in accumulation of paused RNAPII at *S. pombe* downstream pause elements (Aranda and Proudfoot 1999). Another key factor recruited to pericentromeric regions by Seb1 is the remodeling/HDAC (histone deactylase) complex SHREC (Marina et al. 2013). Thus, Seb1-paused RNAPII may promote heterochromatin assembly through multiple mechanisms. Given the tight coupling of this heterochromatin signal to RNAPII activity, it is tempting to speculate that Seb1-mediated pausing may have evolved from a surveillance mechanism for silencing foreign DNA. Finally, pathogenic triplet repeat expansions in the Friedreich ataxia gene *FXN* concomitantly display a block to transcriptional elongation and the appearance of H3K9me on *FXN* (Punga and Bühler 2010; Li et al. 2015), raising the possibility that pause-induced heterochromatin underlies disease pathogenesis.

## Materials and methods

### Yeast strains, plasmids, and media

A list of all *S. pombe* strains and plasmids used in this study is in Supplemental Table S5. Cells were grown at 30°C in synthetic complete (SC) medium with adenine and amino acid supplements with reduced levels of uracil (150 mg/L) for PAR-CLIP or in Edinburgh minimal medium (EMM) supplemented with adenine, uracil, and the appropriate amino acids with or without 15 µM thiamine for NET-seq and ChIP-seq.

### NET-seq

NET-seq experiments were conducted as described previously (Churchman and Weissman 2012) with minor alterations for *S. pombe*. *S. pombe* cultures were grown in 1 L of EMM without thiamine to an OD_600_ of 0.7, harvested via filtration, and flash-frozen in liquid nitrogen. Lysis and immunoprecipitation were conducted as described previously (Shetty et al. 2017). Adaptor ligation was performed using random hexamer-barcoded adaptors. All strains were analyzed in duplicate, and sequencing was conducted on a HiSeq 4000 platform.

### RNA-seq

Strains were grown in YS medium + 3% glucose overnight to OD_600_ = 0.7. Cells were harvested by centrifugation, washed twice with ice-cold water, and flash-frozen. Pellets were resuspended in 1 mL of Trizol (Thermo Fisher Scientific, 15596026). Zirconia-silica beads (0.5-mm; BioSpec, 11079105z) were added, and lysis was accomplished by three cycles of bead beating for 90 sec on high (Bead Ruptor 12 homogenizer, OMNI International). Following centrifugation at 14,000 rpm for 10 min at 4°C, the supernatant was transferred to a microcentrifuge tube, extracted once with chloroform, and precipitated with isopropanol. Following resuspension and reprecipitation with isopropanol, pellets were washed with 75% ethanol and air-dried for 30 min. Pellets were resuspended in 150 µL of RNase-free water.

One microgram of total RNA was used to isolate mRNA using PolyATtract systems III and IV (Promega, Z5310) according to the manufacturer's instructions. Input RNA quality and mRNA purity were verified by Bioanalyzer RNA 6000 Pico kit (Agilent, 5067-1513). To address the issue of genomic DNA contamination in RNA samples, we used Zymo RNA Clean and concentrator kit 5 (Zymo Research, 11-326) according to the manufacturer's instruction. A sequencing library was constructed using NEBNext Ultra Directional RNA library preparation kit for Illumina (New England Biolab, E740S). Libraries were analyzed for quality and average size on Bioanalyzer high-sensitivity DNA kit (Agilent, 5067-4626). The sequencing was performed on an Illumina HiSeq 4000 platform.

### PAR-CLIP

PAR-CLIP experiments were conducted as a combination of PAR-CLIP and CRAC (cross-linking and analysis of cDNA) protocols (Granneman et al. 2009; Hafner et al. 2010) in two replicates. Cells were grown in 2 L of SC medium to an OD_600_ of 0.75. 4-thiouracil (Sigma, 440736-1G) was added to a final concentration of 1.3 mM for 15 min in the dark at 30°C. Samples were immediately cross-linked using the UV Power-Shot handheld UV curing system (SPDI UV) at a 365-nm wavelength for 15 min while continuously stirring. Samples were collected by filtration, resuspended in 6 mL of buffer TMN150 (50 mM Tris-HCl at pH 7.8, 150 mM NaCl, 1.5 mM MgCl_2_, 0.5% NP-40, 5 mM β-mercaptoethanol), and frozen as “yeast popcorn” by drop-wise addition to liquid nitrogen. This material was lysed by ball mill for five cycles at 15 Hz for 3 min (Mixer Mill MM 301, Retsch). CRAC was then performed from this point on as described previously (Granneman et al. 2009) with the exception of gel extraction, which was conducted by electroelution of the gel piece containing the radioactively labeled RNA sample using D-TubeTM dialyzer midi tubes (EMD Millipore, 71507-3). Electroelution was carried out in 1× MOPS SDS PAGE buffer (5 mM MOPS, 50 mM Tris, 0.1% SDS, 1 mM EDTA) and run for 2 h at 100 V. The isolated sample was transferred to a fresh tube, and 100 µg of Proteinase K (Sigma, P2308) was added to the sample. Procedures after Proteinase K treatment were conducted as described previously (Granneman et al. 2009), and samples were sequenced on the HiSeq 4000 platform.

### Spotting assay

Strains were grown overnight to saturation and diluted to an OD_600_ of 1. Serial dilutions were performed with a dilution factor of 5. For *ura4*^+^ silencing assays, cells were grown on nonselective medium and 2 g/L 5-fluoroortic acid (FOA) (unless stated otherwise) YS plates for 3 d at 30°C. For *tfs1*^*DN*^ induction assays, cells were grown on YS, EMM −leu −thiamine, or EMM −leu +thiamine for 3 d.

### ChIP-seq

ChIP-seq was conducted as described previously (Inada et al. 2016). ChIP-seq immunoprecipitations were performed with 10 μg of anti-H3K9me2 (Abcam, ab1220) and 10 µg of anti-Flag (Sigma, P3165). Samples were sequenced on a HiSeq 4000 platform.

### NET-seq analysis: genome alignment

For NET-seq analysis, adapter sequences (using ATCTCGTAT) were removed, and reads were flattened to remove sequence duplicates. Barcoded reads were then mapped to the *S. pombe* genome (Wood et al. 2012) using Bowtie (Langmead et al. 2009) to align and omit any sequence reads that were misprimed during the reverse transcription step of NET-seq and thus lacked a barcode using the following flags: -M1 --best --strata. Unaligned files were collected for further analysis. Barcodes were removed, and the new unique debarcoded reads were realigned to the genome using the following flags in Bowtie: -M1 --best --strata.

### NET-seq analysis: cluster finding

High-density regions of NET-seq signal were defined across centromeres and coding regions to compare NET-seq density between genotypes. First, NET-seq peaks were discovered by calculating robust *Z*-scores (based on median and median absolute deviation) from the log_2_ transform of the number of reads starting at each position in the defined region (centromere fragment or transcript). Positions with a robust *Z*-score of at least 2 and at least 10 unique reads were considered peaks. Next, peaks were clustered together using a sliding window (width = 50, increment = 10). The density of the cluster was calculated as the number of reads in the cluster divided by the size of the cluster in kilobases.

To determine cluster densities for each fragment derived from the right arm of centromere 1, the sum of cluster densities was normalized to the sum of all densities in each sample. Error bars represent the range of two replicates.

### NET-seq analysis: traveling ratio

Traveling ratios were calculated for every nonoverlapping annotated transcript at least 1000 nucleotides (nt) in length according to the Pombase annotation (Wood et al. 2012). Transcripts with <50 total reads were excluded from the analysis. The traveling ratio was determined for a 0.5-kb window either immediately after the TSS (5′ traveling ratio) or immediately before the CPA site (3′ traveling ratio). Transcripts <1 kb in length were omitted from this analysis to ensure that the 0.5-kb 5′ and 3′ windows used for each traveling ratio do not overlap. Reads were counted in this window and across the entire transcript and then divided by the size of the window or transcript, respectively. Transcripts were clustered using *K*-means (sklearn.cluster.Kmeans, three centroids) based on the travelling ratio at each end of the *clr4Δ* and *clr4Δ seb1-1* mutant on one replicate. *P*-values for each cluster for the difference between the *clr4Δ* and *clr4Δ seb1-1* traveling ratio distributions were determined by KS test for each pair of replicates. Traveling ratio cumulative distribution function (CDF) plots were similar between replicates, and a single replicate is presented.

### NET-seq analysis: dwell time

Dwell time was determined by normalizing peak height to the average NET-seq signal density of the surrounding 100 nt. NET-seq peaks with at least a twofold decrease from *clr4Δ* to *clr4Δ seb1-1* in both replicates were considered Seb1-dependent. *P*-values were determined by KS test.

### RNA-seq analysis

Analysis was performed using TopHat (Trapnell et al. 2009) and DESeq2 (Love et al. 2014). Changes in transcript expression levels required a greater than twofold change in mutants compared with wild type to be considered significantly changed enough to have a functional consequence. Data analysis was performed on two replicates per condition.

To determine the fraction of reads derived from the expression of *tfs1*^*DN*^, we divided the total number of reads that specifically aligned to the mutated region of the *tfs1*^*DN*^ allele by the total number of reads (both wild-type and mutant alleles) that aligned to this same region.

### Seb1 PAR-CLIP data analysis by PARalyzer

For PAR-CLIP analysis, adapter sequences were removed, and reads were mapped to the *S. pombe* genome (Wood et al. 2012) using Bowtie (Langmead et al. 2009), allowing for three mismatches with the following flags: -M1 -v3 --best --strata. Seb1-binding site read clusters were identified with PARalyzer (Corcoran et al. 2011). Reads of <20 nt were omitted, and read clusters required at least 10 reads and at least two T → C conversions per cluster to be called as a Seb1-binding site. The PARalyzer OUTPUTCLUSTERSFILE file was converted to a genome browser readable file (.bam) for analysis. PAR-CLIP cluster coverage was calculated as the fraction of the interval of interest harboring a PARalyzer-called PAR-CLIP cluster (centromere arm, coding gene, or ncRNA). *P*-values were determined by KS test.

### DREME motif analysis

DREME (Bailey 2011) motif discovery for short ungapped sequences was used to find Seb1-specific binding motifs. PAR-CLIP clusters that were flattened to remove identical recurrent sequence clusters originating from all three centromeric regions were subjected to motif analysis. A shuffled sequence set created from the input sequences was used as a control.

### ChIP-seq analysis

ChIP-seq analysis was conducted as described previously (Inada et al. 2016). Briefly, adaptor sequences from ChIP-seq sequencing libraries were removed (using GATCGGAAGA), and reads <20 nt were omitted. Reads were aligned to the *S. pombe* genome (Wood et al. 2012) using Bowtie (Langmead et al. 2009) with the following flags: -M1 --best --strata. Aligned reads were smoothed over a 1-kb window.

### ChIP-seq analysis: PIER discovery

H3K9me ChIP-seq peaks were considered as novel ectopic sites of H3K9me if two criteria were met: (1) H3K9me peaks were threefold or greater than the genome background signal in the isolate, and (2) when normalized to the whole-cell extract (denoted “H3K9me enrichment” in the figures), the H3K9me signal at the peak was threefold or greater higher than the background and parental H3K9me ChIP-seq signals. A curated list of genomic regions previously observed to have a propensity to form heterochromatin in various *S. pombe* backgrounds (Yamanaka et al. 2012; Zofall et al. 2012; Wang et al. 2015) was generated (Supplemental Table S6). In *epe1Δ* backgrounds, oscillation and spreading of H3K9me can occur (Trewick et al. 2007); thus, peaks within 10 kb of our curated list of H3K9me nucleation sites or within 10 kb of H3K9me regions present in the parental strain were not counted as novel H3K9me nucleation events.

### ChIP-seq analysis: H3K9me levels at HOODs, islands, meiotic genes, and PIERs

For all isolates and whole-cell extracts, RPKMs (reads per kilobase transcript per million mapped reads) for each region in Supplemental Table S6 and all PIERs were normalized to the RPKM of a 10-kb window surrounding each region (5 kb upstream and downstream). The ratio of H3K9me enrichment values from isolates to the whole-cell extracts were plotted as a heat map (Supplemental Fig. S8).

### Data sets

All available sequencing data sets are listed in Supplemental Table S7 and were deposited in the Gene Expression Omnibus with the superseries accession number GSE114540.

## Supplementary Material

Supplemental Material

## References

[GAD310136PARC1] Allshire RC, Madhani HD. 2018 Ten principles of heterochromatin formation and function. Nat Rev Mol Cell Biol 19: 229–244. 29235574 10.1038/nrm.2017.119 PMC6822695

[GAD310136PARC2] Aranda A, Proudfoot NJ. 1999 Definition of transcriptional pause elements in fission yeast. Mol Cell Biol 19: 1251–1261. 9891059 10.1128/mcb.19.2.1251 PMC116054

[GAD310136PARC3] Audergon PN, Catania S, Kagansky A, Tong P, Shukla M, Pidoux AL, Allshire RC. 2015 Epigenetics. Restricted epigenetic inheritance of H3K9 methylation. Science 348: 132–135. 25838386 10.1126/science.1260638 PMC4397586

[GAD310136PARC4] Aygun O, Mehta S, Grewal SI. 2013 HDAC-mediated suppression of histone turnover promotes epigenetic stability of heterochromatin. Nat Struct Mol Biol 20: 547–554. 23604080 10.1038/nsmb.2565 PMC3661211

[GAD310136PARC5] Bailey TL. 2011 DREME: motif discovery in transcription factor ChIP-seq data. Bioinformatics 27: 1653–1659. 21543442 10.1093/bioinformatics/btr261 PMC3106199

[GAD310136PARC6] Bannister AJ, Zegerman P, Partridge JF, Miska EA, Thomas JO, Allshire RC, Kouzarides T. 2001 Selective recognition of methylated lysine 9 on histone H3 by the HP1 chromo domain. Nature 410: 120–124. 11242054 10.1038/35065138

[GAD310136PARC7] Bayne EH, White SA, Kagansky A, Bijos DA, Sanchez-Pulido L, Hoe K-L, Kim D-U, Park H-O, Ponting CP, Rappsilber J, 2010 Stc1: a critical link between RNAi and chromatin modification required for heterochromatin integrity. Cell 140: 666–677. 20211136 10.1016/j.cell.2010.01.038 PMC2875855

[GAD310136PARC8] Buhler M, Verdel A, Moazed D. 2006 Tethering RITS to a nascent transcript initiates RNAi- and heterochromatin-dependent gene silencing. Cell 125: 873–886. 16751098 10.1016/j.cell.2006.04.025

[GAD310136PARC9] Chalamcharla VR, Folco HD, Dhakshnamoorthy J, Grewal SI. 2015 Conserved factor Dhp1/Rat1/Xrn2 triggers premature transcription termination and nucleates heterochromatin to promote gene silencing. Proc Natl Acad Sci 112: 15548–15555. 26631744 10.1073/pnas.1522127112 PMC4697380

[GAD310136PARC10] Chan FL, Wong LH. 2012 Transcription in the maintenance of centromere chromatin identity. Nucleic Acids Res 40: 11178–11188. 23066104 10.1093/nar/gks921 PMC3526279

[GAD310136PARC11] Chen ES, Zhang K, Nicolas E, Cam HP, Zofall M, Grewal SI. 2008 Cell cycle control of centromeric repeat transcription and heterochromatin assembly. Nature 451: 734–737. 18216783 10.1038/nature06561

[GAD310136PARC12] Churchman LS, Weissman JS. 2011 Nascent transcript sequencing visualizes transcription at nucleotide resolution. Nature 469: 368–373. 21248844 10.1038/nature09652 PMC3880149

[GAD310136PARC13] Churchman LS, Weissman JS. 2012 Native elongating transcript sequencing (NET-seq). Curr Protoc Mol Biol 98: 14.4.1–14.4.17. 10.1002/0471142727.mb0414s98 22470065

[GAD310136PARC14] Contreras X, Benkirane M, Kiernan R. 2013 Premature termination of transcription by RNAP II. Transcription 4: 72–76. 23714697 10.4161/trns.24148 PMC3646057

[GAD310136PARC15] Corcoran DL, Georgiev S, Mukherjee N, Gottwein E, Skalsky RL, Keene JD, Ohler U. 2011 PARalyzer: definition of RNA binding sites from PAR-CLIP short-read sequence data. Genome Biol 12: R79. 21851591 10.1186/gb-2011-12-8-r79 PMC3302668

[GAD310136PARC16] Dubarry M, Loiodice I, Chen CL, Thermes C, Taddei A. 2011 Tight protein–DNA interactions favor gene silencing. Genes Dev 25: 1365–1370. 21724830 10.1101/gad.611011 PMC3134080

[GAD310136PARC17] Freitag M, Lee DW, Kothe GO, Pratt RJ, Aramayo R, Selker EU. 2004 DNA methylation is independent of RNA interference in Neurospora. Science 304: 1939. 15218142 10.1126/science.1099709

[GAD310136PARC18] Gallagher PS, Larkin M, Thillainadesan G, Dhakshnamoorthy J, Balachandran V, Xiao H, Wellman C, Chatterjee R, Wheeler D, Grewal SIS. 2018 Iron homeostasis regulates facultative heterochromatin assembly in adaptive genome control. Nat Struct Mol Biol 25: 372–383. 29686279 10.1038/s41594-018-0056-2 PMC5936480

[GAD310136PARC19] Granneman S, Kudla G, Petfalski E, Tollervey D. 2009 Identification of protein binding sites on U3 snoRNA and pre-rRNA by UV cross-linking and high-throughput analysis of cDNAs. Proc Natl Acad Sci 106: 9613–9618. 19482942 10.1073/pnas.0901997106 PMC2688437

[GAD310136PARC20] Hafner M, Landthaler M, Burger L, Khorshid M, Hausser J, Berninger P, Rothballer A, Ascano M Jr, Jungkamp AC, Munschauer M, 2010 Transcriptome-wide identification of RNA-binding protein and microRNA target sites by PAR-CLIP. Cell 141: 129–141. 20371350 10.1016/j.cell.2010.03.009 PMC2861495

[GAD310136PARC21] Imashimizu M, Kireeva ML, Lubkowska L, Gotte D, Parks AR, Strathern JN, Kashlev M. 2013 Intrinsic translocation barrier as an initial step in pausing by RNA polymerase II. J Mol Biol 425: 697–712. 23238253 10.1016/j.jmb.2012.12.002 PMC7649676

[GAD310136PARC22] Inada M, Nichols RJ, Parsa JY, Homer CM, Benn RA, Hoxie RS, Madhani HD, Shuman S, Schwer B, Pleiss JA. 2016 Phospho-site mutants of the RNA polymerase II C-terminal domain alter subtelomeric gene expression and chromatin modification state in fission yeast. Nucleic Acids Res 44: 9180–9189. 27402158 10.1093/nar/gkw603 PMC5100562

[GAD310136PARC23] Jeon C, Yoon H, Agarwal K. 1994 The transcription factor TFIIS zinc ribbon dipeptide Asp–Glu is critical for stimulation of elongation and RNA cleavage by RNA polymerase II. Proc Natl Acad Sci 91: 9106–9110. 8090778 10.1073/pnas.91.19.9106 PMC44756

[GAD310136PARC24] Kloc A, Martienssen R. 2008 RNAi, heterochromatin and the cell cycle. Trends Genet 24: 511–517. 18778867 10.1016/j.tig.2008.08.002

[GAD310136PARC25] Kowalik KM, Shimada Y, Flury V, Stadler MB, Batki J, Buhler M. 2015 The Paf1 complex represses small-RNA-mediated epigenetic gene silencing. Nature 520: 248–252. 25807481 10.1038/nature14337 PMC4398878

[GAD310136PARC26] Lachner M, O'Carroll D, Rea S, Mechtler K, Jenuwein T. 2001 Methylation of histone H3 lysine 9 creates a binding site for HP1 proteins. Nature 410: 116–120. 11242053 10.1038/35065132

[GAD310136PARC27] Langmead B, Trapnell C, Pop M, Salzberg SL. 2009 Ultrafast and memory-efficient alignment of short DNA sequences to the human genome. Genome Biol 10: R25. 19261174 10.1186/gb-2009-10-3-r25 PMC2690996

[GAD310136PARC28] Larson MH, Mooney RA, Peters JM, Windgassen T, Nayak D, Gross CA, Block SM, Greenleaf WJ, Landick R, Weissman JS. 2014 A pause sequence enriched at translation start sites drives transcription dynamics in vivo. Science 344: 1042–1047. 24789973 10.1126/science.1251871 PMC4108260

[GAD310136PARC29] Lemay JF, Larochelle M, Marguerat S, Atkinson S, Bahler J, Bachand F. 2014 The RNA exosome promotes transcription termination of backtracked RNA polymerase II. Nat Struct Mol Biol 21: 919–926. 25240800 10.1038/nsmb.2893

[GAD310136PARC30] Lemay JF, Marguerat S, Larochelle M, Liu X, van Nues R, Hunyadkurti J, Hoque M, Tian B, Granneman S, Bahler J, 2016 The Nrd1-like protein Seb1 coordinates cotranscriptional 3′ end processing and polyadenylation site selection. Genes Dev 30: 1558–1572. 27401558 10.1101/gad.280222.116 PMC4949328

[GAD310136PARC31] Li F, Martienssen R, Cande WZ. 2011 Coordination of DNA replication and histone modification by the Rik1–Dos2 complex. Nature 475: 244–248. 21725325 10.1038/nature10161 PMC3163161

[GAD310136PARC32] Li Y, Lu Y, Polak U, Lin K, Shen J, Farmer J, Seyer L, Bhalla AD, Rozwadowska N, Lynch DR, 2015 Expanded GAA repeats impede transcription elongation through the FXN gene and induce transcriptional silencing that is restricted to the FXN locus. Hum Mol Genet 24: 6932–6943. 26401053 10.1093/hmg/ddv397 PMC4654050

[GAD310136PARC33] Li W, Yi J, Agbu P, Zhou Z, Kelley RL, Kallgren S, Jia S, He X. 2017 Replication stress affects the fidelity of nucleosome-mediated epigenetic inheritance. PLoS Genet 13: e1006900. 28749973 10.1371/journal.pgen.1006900 PMC5549764

[GAD310136PARC34] Love MI, Huber W, Anders S. 2014 Moderated estimation of fold change and dispersion for RNA-seq data with DESeq2. Genome Biol 15: 550. 25516281 10.1186/s13059-014-0550-8 PMC4302049

[GAD310136PARC35] Luna R, Rondón AG, Aguilera A. 2012 New clues to understand the role of THO and other functionally related factors in mRNP biogenesis. Biochim Biophys Acta 1819: 514–520. 22207203 10.1016/j.bbagrm.2011.11.012

[GAD310136PARC36] Marina DB, Shankar S, Natarajan P, Finn KJ, Madhani HD. 2013 A conserved ncRNA-binding protein recruits silencing factors to heterochromatin through an RNAi-independent mechanism. Genes Dev 27: 1851–1856. 24013500 10.1101/gad.226019.113 PMC3778239

[GAD310136PARC37] Matsuyama A, Shirai A, Yashiroda Y, Kamata A, Horinouchi S, Yoshida M. 2004 pDUAL, a multipurpose, multicopy vector capable of chromosomal integration in fission yeast. Yeast 21: 1289–1305. 15546162 10.1002/yea.1181

[GAD310136PARC38] Mayer A, Landry HM, Churchman LS. 2017 Pause & go: from the discovery of RNA polymerase pausing to its functional implications. Curr Opin Cell Biol 46: 72–80. 28363125 10.1016/j.ceb.2017.03.002 PMC5505790

[GAD310136PARC39] Motamedi MR, Verdel A, Colmenares SU, Gerber SA, Gygi SP, Moazed D. 2004 Two RNAi complexes, RITS and RDRC, physically interact and localize to noncoding centromeric RNAs. Cell 119: 789–802. 15607976 10.1016/j.cell.2004.11.034

[GAD310136PARC40] Noma K, Sugiyama T, Cam H, Verdel A, Zofall M, Jia S, Moazed D, Grewal SI. 2004 RITS acts in *cis* to promote RNA interference-mediated transcriptional and post-transcriptional silencing. Nat Genet 36: 1174–1180. 15475954 10.1038/ng1452

[GAD310136PARC41] Partridge JF, Scott KS, Bannister AJ, Kouzarides T, Allshire RC. 2002 *Cis*-acting DNA from fission yeast centromeres mediates histone H3 methylation and recruitment of silencing factors and cohesin to an ectopic site. Curr Biol 12: 1652–1660. 12361567 10.1016/s0960-9822(02)01177-6

[GAD310136PARC42] Poli J, Gerhold CB, Tosi A, Hustedt N, Seeber A, Sack R, Herzog F, Pasero P, Shimada K, Hopfner KP, 2016 Mec1, INO80, and the PAF1 complex cooperate to limit transcription replication conflicts through RNAPII removal during replication stress. Genes Dev 30: 337–354. 26798134 10.1101/gad.273813.115 PMC4743062

[GAD310136PARC43] Punga T, Bühler M. 2010 Long intronic GAA repeats causing Friedreich ataxia impede transcription elongation. EMBO Mol Med 2: 120–129. 20373285 10.1002/emmm.201000064 PMC3377279

[GAD310136PARC44] Rea S, Eisenhaber F, O'Carroll D, Strahl BD, Sun ZW, Schmid M, Opravil S, Mechtler K, Ponting CP, Allis CD, 2000 Regulation of chromatin structure by site-specific histone H3 methyltransferases. Nature 406: 593–599. 10949293 10.1038/35020506

[GAD310136PARC45] Reyes-Turcu FE, Zhang K, Zofall M, Chen E, Grewal SI. 2011 Defects in RNA quality control factors reveal RNAi-independent nucleation of heterochromatin. Nat Struct Mol Biol 18: 1132–1138. 21892171 10.1038/nsmb.2122 PMC3190054

[GAD310136PARC46] Sadaie M, Iida T, Urano T, Nakayama J. 2004 A chromodomain protein, Chp1, is required for the establishment of heterochromatin in fission yeast. EMBO J 23: 3825–3835. 15372076 10.1038/sj.emboj.7600401 PMC522800

[GAD310136PARC47] Sadeghi L, Prasad P, Ekwall K, Cohen A, Svensson JP. 2015 The Paf1 complex factors Leo1 and Paf1 promote local histone turnover to modulate chromatin states in fission yeast. EMBO Rep 16: 1673–1687. 26518661 10.15252/embr.201541214 PMC4687421

[GAD310136PARC48] Shetty A, Kallgren SP, Demel C, Maier KC, Spatt D, Alver BH, Cramer P, Park PJ, Winston F. 2017 Spt5 plays vital roles in the control of sense and Antisense transcription elongation. Mol Cell 66: 77–88.e5. 28366642 10.1016/j.molcel.2017.02.023 PMC5394798

[GAD310136PARC49] Sigurdsson S, Dirac-Svejstrup AB, Svejstrup JQ. 2010 Evidence that transcript cleavage is essential for RNA polymerase II transcription and cell viability. Mol Cell 38: 202–210. 20417599 10.1016/j.molcel.2010.02.026 PMC2994637

[GAD310136PARC50] Simmer F, Buscaino A, Kos-Braun IC, Kagansky A, Boukaba A, Urano T, Kerr ARW, Allshire RC. 2010 Hairpin RNA induces secondary small interfering RNA synthesis and silencing in *trans* in fission yeast. EMBO Rep 11: 112–118. 20062003 10.1038/embor.2009.273 PMC2828748

[GAD310136PARC51] Singh G, Klar AJS. 2008 Mutations in deoxyribonucleotide biosynthesis pathway cause spreading of silencing across heterochromatic barriers at the mating-type region of the fission yeast. Yeast 25: 117–128. 18030666 10.1002/yea.1569

[GAD310136PARC52] Sugiyama T, Cam H, Verdel A, Moazed D, Grewal SI. 2005 RNA-dependent RNA polymerase is an essential component of a self-enforcing loop coupling heterochromatin assembly to siRNA production. Proc Natl Acad Sci 102: 152–157. 15615848 10.1073/pnas.0407641102 PMC544066

[GAD310136PARC53] Sugiyama T, Cam HP, Sugiyama R, Noma K, Zofall M, Kobayashi R, Grewal SI. 2007 SHREC, an effector complex for heterochromatic transcriptional silencing. Cell 128: 491–504. 17289569 10.1016/j.cell.2006.12.035

[GAD310136PARC54] Trapnell C, Pachter L, Salzberg SL. 2009 TopHat: discovering splice junctions with RNA-seq. Bioinformatics 25: 1105–1111. 19289445 10.1093/bioinformatics/btp120 PMC2672628

[GAD310136PARC55] Trewick SC, Minc E, Antonelli R, Urano T, Allshire RC. 2007 The JmjC domain protein Epe1 prevents unregulated assembly and disassembly of heterochromatin. EMBO J 26: 4670–4682. 17948055 10.1038/sj.emboj.7601892 PMC2048757

[GAD310136PARC56] Tucker JF, Ohle C, Schermann G, Bendrin K, Zhang W, Fischer T, Zhang K. 2016 A novel epigenetic silencing pathway involving the highly conserved 5′–3′ exoribonuclease Dhp1/Rat1/Xrn2 in *Schizosaccharomyces pombe*. PLoS Genet 12: e1005873. 26889830 10.1371/journal.pgen.1005873 PMC4758730

[GAD310136PARC57] Verdel A, Jia S, Gerber S, Sugiyama T, Gygi S, Grewal SI, Moazed D. 2004 RNAi-mediated targeting of heterochromatin by the RITS complex. Science 303: 672–676. 14704433 10.1126/science.1093686 PMC3244756

[GAD310136PARC58] Verrier L, Taglini F, Barrales RR, Webb S, Urano T, Braun S, Bayne EH. 2015 Global regulation of heterochromatin spreading by Leo1. Open Biol 5: 150045. 25972440 10.1098/rsob.150045 PMC4450266

[GAD310136PARC59] Volpe TA, Kidner C, Hall IM, Teng G, Grewal SI, Martienssen RA. 2002 Regulation of heterochromatic silencing and histone H3 lysine-9 methylation by RNAi. Science 297: 1833–1837. 12193640 10.1126/science.1074973

[GAD310136PARC60] Volpe T, Schramke V, Hamilton GL, White SA, Teng G, Martienssen RA, Allshire RC. 2003 RNA interference is required for normal centromere function in fission yeast. Chromosome Res 11: 137–146. 12733640 10.1023/a:1022815931524

[GAD310136PARC61] Wagschal A, Rousset E, Basavarajaiah P, Contreras X, Harwig A, Laurent-Chabalier S, Nakamura M, Chen X, Zhang K, Meziane O, 2012 Microprocessor, Setx, Xrn2, and Rrp6 co-operate to induce premature termination of transcription by RNAPII. Cell 150: 1147–1157. 22980978 10.1016/j.cell.2012.08.004 PMC3595997

[GAD310136PARC62] Wang SW, Asakawa K, Win TZ, Toda T, Norbury CJ. 2005 Inactivation of the pre-mRNA cleavage and polyadenylation factor Pfs2 in fission yeast causes lethal cell cycle defects. Mol Cell Biol 25: 2288–2296. 15743824 10.1128/MCB.25.6.2288-2296.2005 PMC1061621

[GAD310136PARC63] Wang X, Hsueh YP, Li W, Floyd A, Skalsky R, Heitman J. 2010 Sex-induced silencing defends the genome of *Cryptococcus neoformans* via RNAi. Genes Dev 24: 2566–2582. 21078820 10.1101/gad.1970910 PMC2975932

[GAD310136PARC64] Wang J, Reddy BD, Jia S. 2015 Rapid epigenetic adaptation to uncontrolled heterochromatin spreading. Elife 4: e06179. 10.7554/eLife.06179 PMC4395908 25774602

[GAD310136PARC65] Wittmann S, Renner M, Watts BR, Adams O, Huseyin M, Baejen C, El Omari K, Kilchert C, Heo DH, Kecman T, 2017 The conserved protein Seb1 drives transcription termination by binding RNA polymerase II and nascent RNA. Nat Commun 8: 14861. 28367989 10.1038/ncomms14861 PMC5382271

[GAD310136PARC66] Wood V, Harris MA, McDowall MD, Rutherford K, Vaughan BW, Staines DM, Aslett M, Lock A, Bahler J, Kersey PJ, 2012 PomBase: a comprehensive online resource for fission yeast. Nucleic Acids Res 40: D695–D699. 22039153 10.1093/nar/gkr853 PMC3245111

[GAD310136PARC67] Xu Y, Bernecky C, Lee CT, Maier KC, Schwalb B, Tegunov D, Plitzko JM, Urlaub H, Cramer P. 2017 Architecture of the RNA polymerase II-Paf1C-TFIIS transcription elongation complex. Nat Commun 8: 15741. 10.1038/ncomms15741 PMC5467213 28585565

[GAD310136PARC68] Yamanaka S, Mehta S, Reyes-Turcu FE, Zhuang F, Fuchs RT, Rong Y, Robb GB, Grewal SIS. 2012 RNAi triggered by specialized machinery silences developmental genes and retrotransposons. Nature 493: 557–560. 23151475 10.1038/nature11716 PMC3554839

[GAD310136PARC69] Yamanaka S, Mehta S, Reyes-Turcu FE, Zhuang F, Fuchs RT, Rong Y, Robb GB, Grewal SI. 2013 RNAi triggered by specialized machinery silences developmental genes and retrotransposons. Nature 493: 557–560. 23151475 10.1038/nature11716 PMC3554839

[GAD310136PARC70] Yu R, Jih G, Iglesias N, Moazed D. 2014 Determinants of heterochromatic siRNA biogenesis and function. Mol Cell 53: 262–276. 24374313 10.1016/j.molcel.2013.11.014 PMC4357591

[GAD310136PARC71] Zaratiegui M, Castel SE, Irvine DV, Kloc A, Ren J, Li F, de Castro E, Marin L, Chang AY, Goto D, 2011 RNAi promotes heterochromatic silencing through replication-coupled release of RNA Pol II. Nature 479: 135–138. 22002604 10.1038/nature10501 PMC3391703

[GAD310136PARC72] Zofall M, Grewal SI. 2006 Swi6/HP1 recruits a JmjC domain protein to facilitate transcription of heterochromatic repeats. Mol Cell 22: 681–692. 16762840 10.1016/j.molcel.2006.05.010

[GAD310136PARC73] Zofall M, Yamanaka S, Reyes-Turcu FE, Zhang K, Rubin C, Grewal SI. 2012 RNA elimination machinery targeting meiotic mRNAs promotes facultative heterochromatin formation. Science 335: 96–100. 22144463 10.1126/science.1211651 PMC6338074

