# Supplementary material for: Polymerase pausing induced by sequence-specific RNA-binding protein drives heterochromatin assembly
Source: Genes Dev. 2018 Jul 1;32(13-14):953–64. doi: 10.1101/gad.310136.117 (PMC6075038; doi:10.1101/gad.310136.117)
Supplement: Supplemental Material [file supp_32_13-14_953__index.html]

Polymerase pausing induced by sequence-specific RNA-binding protein drives heterochromatin assembly — Supplemental Material 

# Polymerase pausing induced by sequence-specific RNA-binding protein drives heterochromatin assembly

## Supplemental Material

- Supplemental\_Data\_Itemized\_list.pdf
- Supplemental\_Fig\_S7.pdf
- Supplemental\_Fig\_S11.pdf
- Supplemental\_Table\_S5.pdf
- Supplemental\_Table\_S7.pdf
- Supplemental\_Fig\_S5.pdf
- Supplemental\_Fig\_S9.pdf
- Supplemental\_Table\_S3.pdf
- Supplemental\_Fig\_S3.pdf
- Supplemental\_Table\_S1.xlsx
- Supplemental\_Fig\_S1.pdf
- Supplemental\_Fig\_S8.pdf
- Supplemental\_Fig\_S12.pdf
- Supplemental\_Fig\_S6.pdf
- Supplemental\_Fig\_S10.pdf
- Supplemental\_Table\_S4.xlsx
- Supplemental\_Table\_S6.pdf
- Supplemental\_Fig\_S4.pdf
- Supplemental\_Table\_S2.xlsx
- Supplemental\_Fig\_S2.pdf
- Supplemental\_Fig\_S13.pdf
