# Supplementary material for: Polymerase pausing induced by sequence-specific RNA-binding protein drives heterochromatin assembly
Source: Genes Dev. 2018 Jul 1;32(13-14):953–64. doi: 10.1101/gad.310136.117 (PMC6075038; doi:10.1101/gad.310136.117)
Supplement: Supplemental Material [file supp_32.13-14.953_Supplemental_Data_Itemized_list.pdf]

Supplemental Data

Polymerase pausing induced by sequence-specific RNA binding protein drives heterochromatin assembly

Jahan-Yar Parsa, Selim Boudoukha, Jordan Burke, Christina Homer &
Hiten D. Madhani

Dept. of Biochemistry and Biophysics
University of California
San Francisco, CA 94158

Supplemental Figure S1. *seb1-1* mutant displays statistically significant changes in 5' and 3' RNAPII traveling ratios.
Supplement to Figure 1. Statistics of NET-Seq traveling ratios.

Supplemental Figure S2. RNA-seq anlaysis of *seb1-1.*
Supplement to Figure 2.

Supplemental Figure S3. PAR-CLIP analysis of Seb1 at all three centromeres.
Supplement to Figure 2. Replicate Seb1 PAR-CLIP data for all centromeres.

Supplemental Figure S4. Seb1 induces RNAPII pauses with long dwell times throughout the genome.
Supplement to Figure 2. Replicate data for Figures 2 C, D, and E.

Supplemental Figure S5. Seb1 induces pauses within centromere fragments that have a capacity to assemble heterochromatin.
Supplement to Figure 2. Close up of NET-seq signals in centromere fragments.

Supplemental Figure S6. ***pnmt1$^+$-tfs1^{DN}*** **expression impacts traveling ratios genome wide.**

Supplement to Figure 3. TFIIS^{DN} is functional and expressed.

Supplemental Figure S7. TFIIS^{DN} expression induces ectopic heterochromatin.

Supplement to Figure 3. Genome wide images of isolates with PIERs.

Supplemental Figure S8. H3K9me enrichment at known heterochromatin nucleation sites and PIERs for all strains.

Supplement to Figures 3 and 4.

Supplemental Figure S9. TFIIS^{DN} expression silences genes within a PIER but does not alter expression of heterochromatin factors.

Supplement to Figure 3.

Supplemental Figure S10. ***epe1Δ ago1Δ*** **strains can adaptively silence the** ***clr4$^+$*** **locus.**

Supplement to Figure 4.

Supplemental Figure S11. PIER formation is RNAi-independent and expression of TFIIS^{DN} can rescue the adaptive silencing of ***clr4$^+$*** **observed in** ***epe1Δ ago1Δ*** **strains.**

Supplement to Figure 4. Genome wide images of isolates with PIERs.

Supplemental Figure S12. PIERs require Seb1 for assembly and enrichment of the H3K9me signal.

Supplement to Figure 5.

Supplemental Figure S13. Mutations in CPA machinery result in increased H3K9me enrichment at constitutive heterochromatic loci.
Supplement to Figure 5.

Supplemental Table S1. RNA-Seq analysis of *seb1-1* vs WT transcriptomes.

Supplemental Table S2. NET-seq peak clusters identified in *clr4*Δ at centromeres and examined for their presence in *clr4*Δ *seb1-1*.

Supplemental Table S3. List of chromosome coordinates of the fragments used in this study from the right arm of centromere I.

Supplemental Table S4. RNA-Seq analysis of *epe1*Δ *tfs1*^{DN} vs *epe1*Δ transcriptomes.

Supplemental Table S5. List of strains and plasmids used in this study.

Supplemental Table S6. Known heterochromatin recruitment sites.

Supplemental Table S7. Table of data sets used in this study and associated GEO accession numbers.
