# Supplementary material for: Polymerase pausing induced by sequence-specific RNA-binding protein drives heterochromatin assembly
Source: Genes Dev. 2018 Jul 1;32(13-14):953–64. doi: 10.1101/gad.310136.117 (PMC6075038; doi:10.1101/gad.310136.117)
Supplement: Supplemental Material [file supp_32.13-14.953_Supplemental_Fig_S7.pdf]

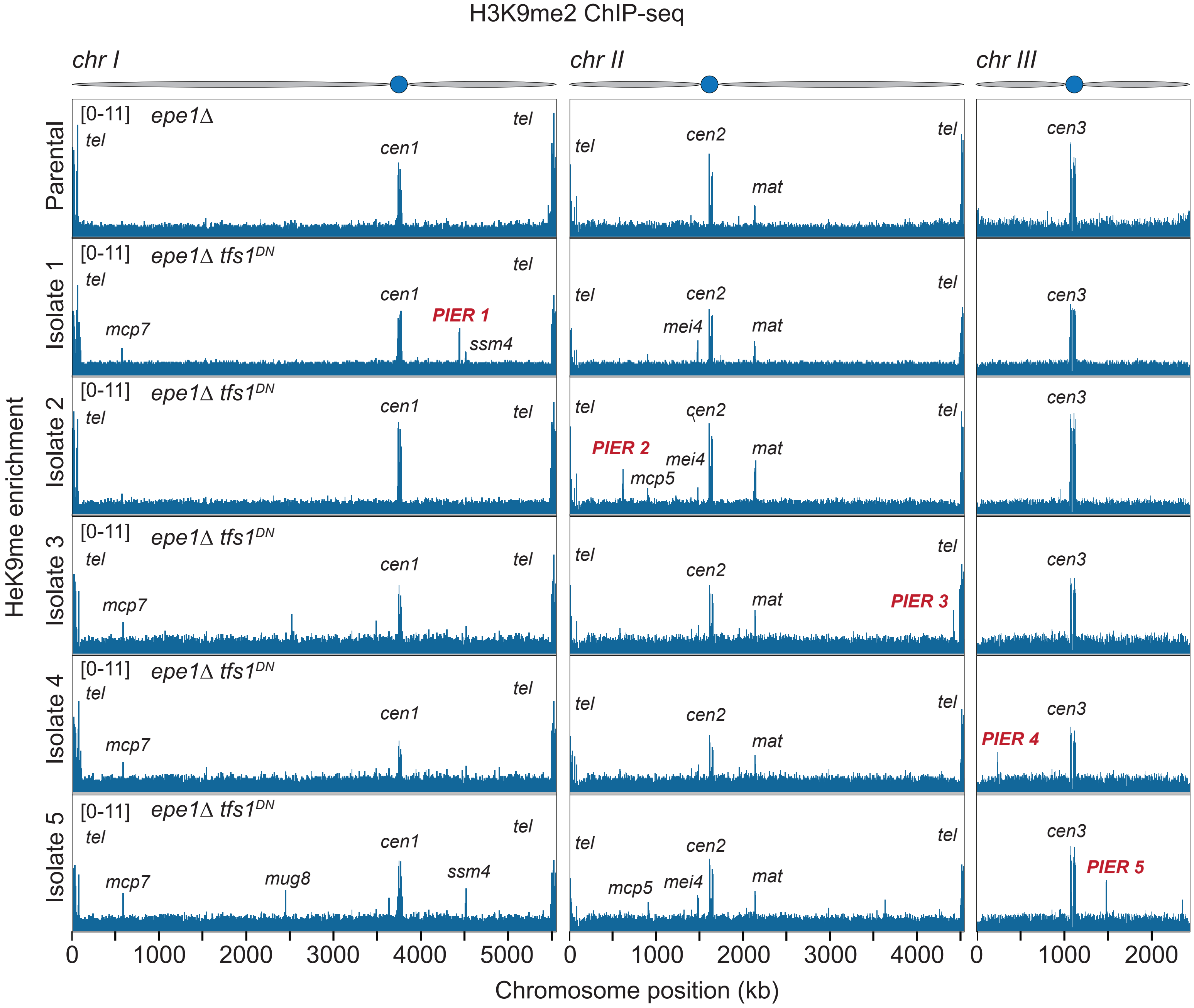

Supplemental Figure S7. TFIIS^{DN} expression induces ectopic heterochromatin. Whole genome plots of H3K9me2 ChIP-seq enrichment for parental (*epe1Δ*) and *epe1Δ tfs1^{DN}* isolates 1 through 5. Locations of PIERs noted in each plot.
