# Supplementary material for: Polymerase pausing induced by sequence-specific RNA-binding protein drives heterochromatin assembly
Source: Genes Dev. 2018 Jul 1;32(13-14):953–64. doi: 10.1101/gad.310136.117 (PMC6075038; doi:10.1101/gad.310136.117)
Supplement: Supplemental Material [file supp_32.13-14.953_Supplemental_Fig_S11.pdf]

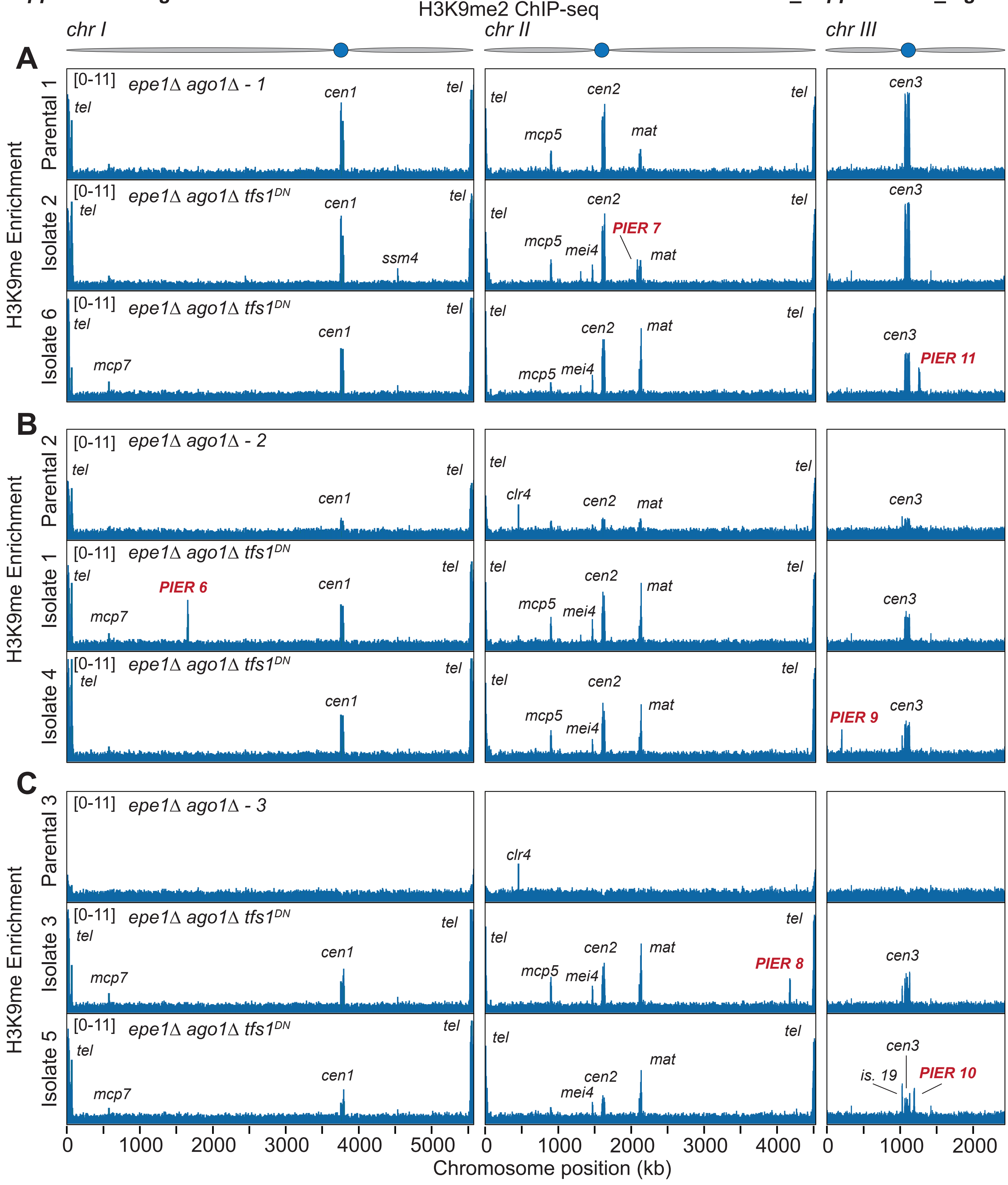
H3K9me2 ChIP-seq
chr I
chr II
chr III
A
B
C
H3K9me Enrichment
Parental 1
Isolate 2
Isolate 6
Parental 2
Isolate 1
Isolate 4
Parental 3
Isolate 3
Isolate 5
[0-11]
epe1Δ ago1Δ - 1
epe1Δ ago1Δ tfs1DN
epe1Δ ago1Δ - 2
epe1Δ ago1Δ - 3
tel
cen1
cen2
cen3
mcp5
mcp7
mei4
mat
ssm4
clr4
PIER 6
PIER 7
PIER 8
PIER 9
PIER 10
PIER 11
is. 19
0
1000
2000
3000
4000
5000
Chromosome position (kb)

Supplemental Figure S11. PIER formation is RNAi-independent and expression of TFIIS^{DN} can rescue the adaptive silencing of *clr4*$^{+}$ observed in *epe1*Δ *ago1*Δ strains. A-C) Genome wide plots of H3K9me2 enrichment. **A)** Parental (*epe1*Δ *ago1*Δ*-1*) and two independent isolates of *epe1*Δ *ago1*Δ *tfs1*^{DN} derived from this parental are plotted depicting PIERs 7 and 11. **B)** Parental (*epe1*Δ *ago1*Δ*-2*), initially harboring H3K9me2 at *clr4*$^{+}$, and two independent isolates of *epe1*Δ *ago1*Δ *tfs1*^{DN} derived from this parental are plotted depicting PIERs 6 and 9. **C)** Parental (*epe1*Δ *ago1*Δ*-3*), initially harboring H3K9me2 at *clr4*$^{+}$, and two independent isolates of *epe1*Δ *ago1*Δ *tfs1*^{DN} derived from this parental are plotted depicting PIERs 8 and 10.
