# Supplementary material for: Polymerase pausing induced by sequence-specific RNA-binding protein drives heterochromatin assembly
Source: Genes Dev. 2018 Jul 1;32(13-14):953–64. doi: 10.1101/gad.310136.117 (PMC6075038; doi:10.1101/gad.310136.117)
Supplement: Supplemental Material [file supp_32.13-14.953_Supplemental_Table_S5.pdf]

Supplemental Table S5. List of strains and plasmids used in this study.

Strain Number	Genotype	Use
PM06	*S. pombe* wild type strain 972 *M(h-)*	Parental strain
PM26	PM06, *ura4::padh1*$^+$-Fragment 1A-ter-Bboxes-*natMX*	*ura4*$^+$ Silencing Assay
PM27	PM06, *ura4::padh1*$^+$-Fragment 1S-ter-Bboxes-*natMX*	*ura4*$^+$ Silencing Assay
PM28	PM06, *ura4::padh1*$^+$-Fragment 2A-ter-Bboxes-*natMX*	*ura4*$^+$ Silencing Assay
PM31	PM06, *ura4::padh1*$^+$-Fragment 2S-ter-Bboxes-*natMX*	*ura4*$^+$ Silencing Assay
PM68	PM06, *ura4::padh1*$^+$-Fragment 3A-ter-Bboxes-*natMX*	*ura4*$^+$ Silencing Assay
PM34	PM06, *ura4::padh1*$^+$-Fragment 3S-ter-Bboxes-*natMX*	*ura4*$^+$ Silencing Assay
PM37	PM06, *ura4::padh1*$^+$-Fragment 4A-ter-Bboxes-*natMX*	*ura4*$^+$ Silencing Assay
PM39	PM06, *ura4::padh1*$^+$-Fragment 4S-ter-Bboxes-*natMX*	*ura4*$^+$ Silencing Assay
PM42	PM06, *ura4::padh1*$^+$-Fragment 5A-ter-Bboxes-*natMX*	*ura4*$^+$ Silencing Assay
PM45	PM06, *ura4::padh1*$^+$-Fragment 5S-ter-Bboxes-*natMX*	*ura4*$^+$ Silencing Assay
PM48	PM06, *ura4::padh1*$^+$-Fragment 6A-ter-Bboxes-*natMX*	*ura4*$^+$ Silencing Assay
PM51	PM06, *ura4::padh1*$^+$-Fragment 6S-ter-Bboxes-*natMX*	*ura4*$^+$ Silencing Assay
PM54	PM06, *ura4::padh1*$^+$-Fragment 7A-ter-Bboxes-*natMX*	*ura4*$^+$ Silencing Assay
PM57	PM06, *ura4::padh1*$^+$-Fragment 7S-ter-Bboxes-*natMX*	*ura4*$^+$ Silencing Assay
PM60	PM06, *ura4::padh1*$^+$-Fragment 8A-ter-Bboxes-*natMX*	*ura4*$^+$ Silencing Assay
PM63	PM06, *ura4::padh1*$^+$-Fragment 8S-ter-Bboxes-*natMX*	*ura4*$^+$ Silencing Assay
PM70	PM06, *ura4::padh1*$^+$-Fragment 9A-ter-Bboxes-*natMX*	*ura4*$^+$ Silencing Assay
PM65	PM06, *ura4::padh1*$^+$-Fragment 9S-ter-Bboxes-*natMX*	*ura4*$^+$ Silencing Assay
PM251	*P(h+), ura4-DS/E, ade6-210, leu1-32, imr1L(NcoI)::ura4+, otr1R(SphI)::ade6+*	*ura4*$^+$ Silencing Assay
PM131	PM06, *ura4::padh1*$^+$-ter-Bboxes-*natMX*	*ura4*$^+$ Silencing Assay
PM1316	PM06, *seb1-1::natMX*	RNA-seq
PM2366	*ade6-M210, leu1-32, ura4-DS/E, imr1L(NcoI)::ura4+, clr4::hygMX, rpb3-3xFLAG::kanMX*	NET-Seq Strain and Rpb3-3xFLAG ChIP-Seq
PM2368	PM2368, *seb1-1::natMX*	NET-Seq Strain and Rpb3-3xFLAG ChIP-Seq
PM2369	PM2368, *leu1::pDUAL-pnmt1*$^+$-*tfs1*^{DN}	NET-Seq Strain 1
PM2370	PM2369, *leu1::pDUAL-pnmt1*$^+$-*tfs1*^{DN}	NET-Seq Strain 2
PM2359	*leu1-32, ade6-M210, seb1-cHTP::kanMX, clr4::hygMX*	PAR-CLIP strain
PM964	*ade6-M210, leu1-32, ura4-D18, epe1::kanMX M(h-)*	*epe1*Δ Parental
PM2371	PM964, *leu1::pDUAL-pnmt1*$^+$	Isolate 1
PM2372	PM964, *leu1::pDUAL-pnmt1*$^+$	Isolate 2
PM2373	PM964, *leu1::pDUAL-pnmt1*$^+$	Isolate 3
PM2374	PM964, *leu1::pDUAL-pnmt1*$^+$	Isolate 4
PM2375	PM964, *leu1::pDUAL-pnmt1*$^+$	Isolate 5
PM2376	PM964, *leu1::pDUAL-pnmt1*$^+$	Isolate 6
PM2377	PM964, *leu1::pDUAL-pnmt1*$^+$	Isolate 7
PM2378	PM964, *leu1::pDUAL-pnmt1*$^+$	Isolate 8
PM2379	PM964, *leu1::pDUAL-pnmt1*$^+$	Isolate 9
PM2380	PM964, *leu1::pDUAL-pnmt1*$^+$	Isolate 10
PM2381	PM964, *leu1::pDUAL-pnmt1*$^+$	Isolate 11
PM2382	PM964, *leu1::pDUAL-pnmt1*$^+$	Isolate 12
PM2383	PM964, *leu1::pDUAL-pnmt1*$^+$	Isolate 13
PM2384	PM964, *leu1::pDUAL-pnmt1*$^+$	Isolate 14
PM2385	PM964, *leu1::pDUAL-pnmt1*$^+$	Isolate 15
PM2390	PM964, *leu1::pDUAL-pnmt1*$^+$-*tfs1*^{DN}	Isolate 1 - PIER1
PM2391	PM964, *leu1::pDUAL-pnmt1*$^+$-*tfs1*^{DN}	Isolate 2 - PIER2
PM2392	PM964, *leu1::pDUAL-pnmt1*$^+$-*tfs1*^{DN}	Isolate 3 - PIER3
PM2393	PM964, *leu1::pDUAL-pnmt1*$^+$-*tfs1*^{DN}	Isolate 4 - PIER4

PM2394	PM964, *leu1::pDUAL-pnmt1*$^{+}$*-tfs1*^{DN}	Isolate 5 - PIER5
PM2395	PM964, *leu1::pDUAL-pnmt1*$^{+}$*-tfs1*^{DN}	Isolate 6
PM2396	PM964, *leu1::pDUAL-pnmt1*$^{+}$*-tfs1*^{DN}	Isolate 7
PM2397	PM964, *leu1::pDUAL-pnmt1*$^{+}$*-tfs1*^{DN}	Isolate 8
PM2398	PM964, *leu1::pDUAL-pnmt1*$^{+}$*-tfs1*^{DN}	Isolate 9
PM2399	PM964, *leu1::pDUAL-pnmt1*$^{+}$*-tfs1*^{DN}	Isolate 10
PM2400	PM964, *leu1::pDUAL-pnmt1*$^{+}$*-tfs1*^{DN}	Isolate 11
PM2401	PM964, *leu1::pDUAL-pnmt1*$^{+}$*-tfs1*^{DN}	Isolate 12
PM2402	PM964, *leu1::pDUAL-pnmt1*$^{+}$*-tfs1*^{DN}	Isolate 13
PM2403	PM964, *ago1::natMX*	Parental Isolate 1
PM2404	PM964, *ago1::natMX*	Parental Isolate 2
PM2405	PM964, *ago1::natMX*	Parental Isolate 3
PM2406	PM2404, *leu1::pDUAL-pnmt1*$^{+}$*-tfs1*^{DN}	Isolate 1 - PIER6
PM2407	PM2403, *leu1::pDUAL-pnmt1*$^{+}$*-tfs1*^{DN}	Isolate 2 - PIER7
PM2408	PM2405, *leu1::pDUAL-pnmt1*$^{+}$*-tfs1*^{DN}	Isolate 3 - PIER8
PM2409	PM2404, *leu1::pDUAL-pnmt1*$^{+}$*-tfs1*^{DN}	Isolate 4 - PIER9
PM2410	PM2405, *leu1::pDUAL-pnmt1*$^{+}$*-tfs1*^{DN}	Isolate 5 - PIER10
PM2411	PM2403, *leu1::pDUAL-pnmt1*$^{+}$*-tfs1*^{DN}	Isolate 6 - PIER11
PM2412	PM2403, *leu1::pDUAL-pnmt1*$^{+}$*-tfs1*^{DN}	Isolate 7
PM2413	PM2404, *leu1::pDUAL-pnmt1*$^{+}$*-tfs1*^{DN}	Isolate 8
PM2414	PM2404, *leu1::pDUAL-pnmt1*$^{+}$*-tfs1*^{DN}	Isolate 9
PM2415	PM2404, *leu1::pDUAL-pnmt1*$^{+}$*-tfs1*^{DN}	Isolate 10
PM2416	PM2405, *leu1::pDUAL-pnmt1*$^{+}$*-tfs1*^{DN}	Isolate 11
PM2417	PM2405, *leu1::pDUAL-pnmt1*$^{+}$*-tfs1*^{DN}	Isolate 12
PM2418	*leu1-32, ura4-D18, seb1-1::hygMX, epe1Δ::kanMX*	Parental
PM2419	PM2418, *leu1::pDUAL-pnmt1*$^{+}$*-tfs1*^{DN}	Isolate 1
PM2420	PM2418, *leu1::pDUAL-pnmt1*$^{+}$*-tfs1*^{DN}	Isolate 2
PM2421	PM2418, *leu1::pDUAL-pnmt1*$^{+}$*-tfs1*^{DN}	Isolate 3
PM2422	PM2418, *leu1::pDUAL-pnmt1*$^{+}$*-tfs1*^{DN}	Isolate 4
PM2423	PM2418, *leu1::pDUAL-pnmt1*$^{+}$*-tfs1*^{DN}	Isolate 5
PM2424	PM2418, *leu1::pDUAL-pnmt1*$^{+}$*-tfs1*^{DN}	Isolate 6
PM2425	PM2418, *leu1::pDUAL-pnmt1*$^{+}$*-tfs1*^{DN}	Isolate 7
PM2426	PM2418, *leu1::pDUAL-pnmt1*$^{+}$*-tfs1*^{DN}	Isolate 8
PM2427	PM2418, *leu1::pDUAL-pnmt1*$^{+}$*-tfs1*^{DN}	Isolate 9
PM2428	PM2418, *leu1::pDUAL-pnmt1*$^{+}$*-tfs1*^{DN}	Isolate 10
PM2429	PM2418, *leu1::pDUAL-pnmt1*$^{+}$*-tfs1*^{DN}	Isolate 11
PM2430	PM2418, *leu1::pDUAL-pnmt1*$^{+}$*-tfs1*^{DN}	Isolate 12
PM2431	PM2418, *leu1::pDUAL-pnmt1*$^{+}$*-tfs1*^{DN}	Isolate 13
PM2432	PM2418, *leu1::pDUAL-pnmt1*$^{+}$*-tfs1*^{DN}	Isolate 14
PM2433	PM2418, *leu1::pDUAL-pnmt1*$^{+}$*-tfs1*^{DN}	Isolate 15
PM2434	*h-*	ChIP-seq *
PM2435	PM2434, *pfs2-11*	ChIP-seq *
PM2436	PM28, *seb1-1::hygMX*	*ura4*$^{+}$ Silencing Assay
PM2437	PM63, *seb1-1::hygMX*	*ura4*$^{+}$ Silencing Assay
PM2438	PM65, *seb1-1::hygMX*	*ura4*$^{+}$ Silencing Assay

**Strain number**	**Plasmid**	
BHM2306	*pDUAL-pnmt1*$^{+}$	**
BHM2308	*pDUAL-pnmt1*$^{+}$*-tfs1*^{DN}	

* Obtained from Chris Norbury (The University of Oxford)
** Obtained from Robin Allshire (The University of Edinburgh)
