# Supplementary material for: Polymerase pausing induced by sequence-specific RNA-binding protein drives heterochromatin assembly
Source: Genes Dev. 2018 Jul 1;32(13-14):953–64. doi: 10.1101/gad.310136.117 (PMC6075038; doi:10.1101/gad.310136.117)
Supplement: Supplemental Material [file supp_32.13-14.953_Supplemental_Table_S7.pdf]

Supplemental Table S7. Table of data sets used in this study and associated GEO accession numbers

All data can be found under GEO SuperSeries ***GSE114540***

Sample	Data Set	Strain	Derived from	GEO accession
1	Seb1 PAR-CLIP Replicate 1	PM2359		GSM3143773
2	Seb1 PAR-CLIP Replicate 2	PM2359		GSM3143774
3	NET-seq *clr4*Δ Replicate 1	PM2366		GSM3143767
4	NET-seq *clr4*Δ Replicate 2	PM2366		GSM3143768
5	NET-seq *clr4*Δ *seb1-1* Replicate 1	PM2368		GSM3143771
6	NET-seq *clr4*Δ *seb1-1* Replicate 2	PM2368		GSM3143772
7	NET-seq *clr4*Δ *tfs1*^{DN} Replicate 1	PM2369		GSM3143769
8	NET-seq *clr4*Δ *tfs1*^{DN} Replicate 2	PM2370		GSM3143770
9	RNA-seq WT Replicate 1	PM06		GSM3143775
10	RNA-seq WT Replicate 2	PM06		GSM3143776
11	RNA-seq *seb1-1* Replicate 1	PM1316	PM06	GSM3143781
12	RNA-seq *seb1-1* Replicate 2	PM1316	PM06	GSM3143782
13	RNA-seq *epe1*Δ Replicate 1	PM964		GSM3143777
14	RNA-seq *epe1*Δ Replicate 2	PM964		GSM3143778
15	RNA-seq *epe1*Δ *tfs1*^{DN} Replicate 1	PM2391	PM964	GSM3143779
16	RNA-seq *epe1*Δ *tfs1*^{DN} Replicate 2	PM2391	PM964	GSM3143780
17	ChIP-seq WCE (for samples 19, 22-32)			GSM3143703
18	ChIP-seq WCE (for samples 20, 21, and 33)			GSM3143687
19	ChIP-seq *epe1*Δ Parental 1 (for samples 22-32)	PM964		GSM3143691
20	ChIP-seq *epe1*Δ Parental 2 (for samples 21 and 33)	PM964		GSM3143688
21	ChIP-Seq *epe1*Δ *tfs1*^{DN} Isolate 1	PM2390	PM964	GSM3143689
22	ChIP-Seq *epe1*Δ *tfs1*^{DN} Isolate 2	PM2391	PM964	GSM3143695
23	ChIP-Seq *epe1*Δ *tfs1*^{DN} Isolate 3	PM2392	PM964	GSM3143696
24	ChIP-Seq *epe1*Δ *tfs1*^{DN} Isolate 4	PM2393	PM964	GSM3143697
25	ChIP-Seq *epe1*Δ *tfs1*^{DN} Isolate 5	PM2394	PM964	GSM3143698
26	ChIP-Seq *epe1*Δ *tfs1*^{DN} Isolate 6	PM2395	PM964	GSM3143699
27	ChIP-Seq *epe1*Δ *tfs1*^{DN} Isolate 7	PM2396	PM964	GSM3143700
28	ChIP-Seq *epe1*Δ *tfs1*^{DN} Isolate 8	PM2397	PM964	GSM3143701
29	ChIP-Seq *epe1*Δ *tfs1*^{DN} Isolate 9	PM2398	PM964	GSM3143702
30	ChIP-Seq *epe1*Δ *tfs1*^{DN} Isolate 10	PM2399	PM964	GSM3143692
31	ChIP-Seq *epe1*Δ *tfs1*^{DN} Isolate 11	PM2400	PM964	GSM3143693
32	ChIP-Seq *epe1*Δ *tfs1*^{DN} Isolate 12	PM2401	PM964	GSM3143694
33	ChIP-Seq *epe1*Δ *tfs1*^{DN} Isolate 13	PM2402	PM964	GSM3143690
34	ChIP-seq WCE (for samples 35-50)			GSM3143742
35	ChIP-seq *epe1*Δ Parental (for samples 36-50)	PM964		GSM3143743
36	ChIP-seq *epe1*Δ vector control Isolate 1	PM2371	PM964	GSM3143727
37	ChIP-seq *epe1*Δ vector control Isolate 2	PM2372	PM964	GSM3143734
38	ChIP-seq *epe1*Δ vector control Isolate 3	PM2373	PM964	GSM3143735
39	ChIP-seq *epe1*Δ vector control Isolate 4	PM2374	PM964	GSM3143736
40	ChIP-seq *epe1*Δ vector control Isolate 5	PM2375	PM964	GSM3143737
41	ChIP-seq *epe1*Δ vector control Isolate 6	PM2376	PM964	GSM3143738
42	ChIP-seq *epe1*Δ vector control Isolate 7	PM2377	PM964	GSM3143739
43	ChIP-seq *epe1*Δ vector control Isolate 8	PM2378	PM964	GSM3143740
44	ChIP-seq *epe1*Δ vector control Isolate 9	PM2379	PM964	GSM3143741
45	ChIP-seq *epe1*Δ vector control Isolate 10	PM2380	PM964	GSM3143728
46	ChIP-seq *epe1*Δ vector control Isolate 11	PM2381	PM964	GSM3143729

47	ChIP-seq *epe1*Δ vector control Isolate 12	PM2382	PM964	GSM3143730
48	ChIP-seq *epe1*Δ vector control Isolate 13	PM2383	PM964	GSM3143731
49	ChIP-seq *epe1*Δ vector control Isolate 14	PM2384	PM964	GSM3143732
50	ChIP-seq *epe1*Δ vector control Isolate 15	PM2385	PM964	GSM3143733
51	ChIP-seq WCE (for samples 54, 58, 62, 63)			GSM3143704
52	ChIP-seq WCE (for samples 55, 57, 60, 64, 65, 66)			GSM3143709
53	ChIP-seq WCE (for samples 56, 59, 61, 67, 68)			GSM3143716
54	ChIP-seq *epe1*Δ *ago1*Δ Parental 1	PM2403	PM964	GSM3143705
55	ChIP-seq *epe1*Δ *ago1*Δ Parental 2	PM2404	PM964	GSM3143710
56	ChIP-seq *epe1*Δ *ago1*Δ Parental 3	PM2405	PM964	GSM3143717
57	ChIP-seq *epe1*Δ *ago1*Δ $tfs1^{DN}$ Isolate 1	PM2406	PM2404	GSM3143711
58	ChIP-seq *epe1*Δ *ago1*Δ $tfs1^{DN}$ Isolate 2	PM2407	PM2403	GSM3143706
59	ChIP-seq *epe1*Δ *ago1*Δ $tfs1^{DN}$ Isolate 3	PM2408	PM2405	GSM3143720
60	ChIP-seq *epe1*Δ *ago1*Δ $tfs1^{DN}$ Isolate 4	PM2409	PM2404	GSM3143713
61	ChIP-seq *epe1*Δ *ago1*Δ $tfs1^{DN}$ Isolate 5	PM2410	PM2405	GSM3143721
62	ChIP-seq *epe1*Δ *ago1*Δ $tfs1^{DN}$ Isolate 6	PM2411	PM2403	GSM3143707
63	ChIP-seq *epe1*Δ *ago1*Δ $tfs1^{DN}$ Isolate 7	PM2412	PM2403	GSM3143708
64	ChIP-seq *epe1*Δ *ago1*Δ $tfs1^{DN}$ Isolate 8	PM2413	PM2404	GSM3143714
65	ChIP-seq *epe1*Δ *ago1*Δ $tfs1^{DN}$ Isolate 9	PM2414	PM2404	GSM3143715
66	ChIP-seq *epe1*Δ *ago1*Δ $tfs1^{DN}$ Isolate 10	PM2415	PM2404	GSM3143712
67	ChIP-seq *epe1*Δ *ago1*Δ $tfs1^{DN}$ Isolate 11	PM2416	PM2405	GSM3143718
68	ChIP-seq *epe1*Δ *ago1*Δ $tfs1^{DN}$ Isolate 12	PM2417	PM2405	GSM3143719
69	ChIP-seq WCE (for samples 70-85)			GSM3143760
70	ChIP-seq *seb1-1 epe1*Δ	PM2418		GSM3143744
71	ChIP-seq *seb1-1 epe1*Δ $tfs1^{DN}$ Isolate 1	PM2419	PM2418	GSM3143745
72	ChIP-seq *seb1-1 epe1*Δ $tfs1^{DN}$ Isolate 2	PM2420	PM2418	GSM3143752
73	ChIP-seq *seb1-1 epe1*Δ $tfs1^{DN}$ Isolate 3	PM2421	PM2418	GSM3143753
74	ChIP-seq *seb1-1 epe1*Δ $tfs1^{DN}$ Isolate 4	PM2422	PM2418	GSM3143754
75	ChIP-seq *seb1-1 epe1*Δ $tfs1^{DN}$ Isolate 5	PM2423	PM2418	GSM3143755
76	ChIP-seq *seb1-1 epe1*Δ $tfs1^{DN}$ Isolate 6	PM2424	PM2418	GSM3143756
77	ChIP-seq *seb1-1 epe1*Δ $tfs1^{DN}$ Isolate 7	PM2425	PM2418	GSM3143757
78	ChIP-seq *seb1-1 epe1*Δ $tfs1^{DN}$ Isolate 8	PM2426	PM2418	GSM3143758
79	ChIP-seq *seb1-1 epe1*Δ $tfs1^{DN}$ Isolate 9	PM2427	PM2418	GSM3143759
80	ChIP-seq *seb1-1 epe1*Δ $tfs1^{DN}$ Isolate 10	PM2428	PM2418	GSM3143746
81	ChIP-seq *seb1-1 epe1*Δ $tfs1^{DN}$ Isolate 11	PM2429	PM2418	GSM3143747
82	ChIP-seq *seb1-1 epe1*Δ $tfs1^{DN}$ Isolate 12	PM2430	PM2418	GSM3143748
83	ChIP-seq *seb1-1 epe1*Δ $tfs1^{DN}$ Isolate 13	PM2431	PM2418	GSM3143749
84	ChIP-seq *seb1-1 epe1*Δ $tfs1^{DN}$ Isolate 14	PM2432	PM2418	GSM3143750
85	ChIP-seq *seb1-1 epe1*Δ $tfs1^{DN}$ Isolate 15	PM2433	PM2418	GSM3143751
86	ChIP-seq WCE (for samples 87-88)			GSM3143762
87	ChIP-seq *pfs2*+ Parent replicate 1	PM2434		GSM3143763
88	ChIP-seq *pfs2-11* replicate 1	PM2435	PM2434	GSM3143761
89	ChIP-Seq WCE (fro samples 90-91)			GSM3143765
90	ChIP-seq *pfs2*+ Parent replicate 2	PM2434		GSM3143766
91	ChIP-seq *pfs2-11* replicate 2	PM2435	PM2434	GSM3143764
92	RNAPII ChIP-seq WCE (for samples 93-96)			GSM3143726
93	RNAPII ChIP-seq *clr4*Δ replicate 1	PM2366		GSM3143722
94	RNAPII ChIP-seq *clr4*Δ replicate 2	PM2366		GSM3143723
95	RNAPII ChIP-seq *clr4*Δ *seb1-1* replicate 1	PM2368	PM2366	GSM3143724
96	RNAPII ChIP-seq *clr4*Δ *seb1-1* replicate 2	PM2368	PM2366	GSM3143725
