# Supplementary material for: Polymerase pausing induced by sequence-specific RNA-binding protein drives heterochromatin assembly
Source: Genes Dev. 2018 Jul 1;32(13-14):953–64. doi: 10.1101/gad.310136.117 (PMC6075038; doi:10.1101/gad.310136.117)
Supplement: Supplemental Material [file supp_32.13-14.953_Supplemental_Fig_S5.pdf]

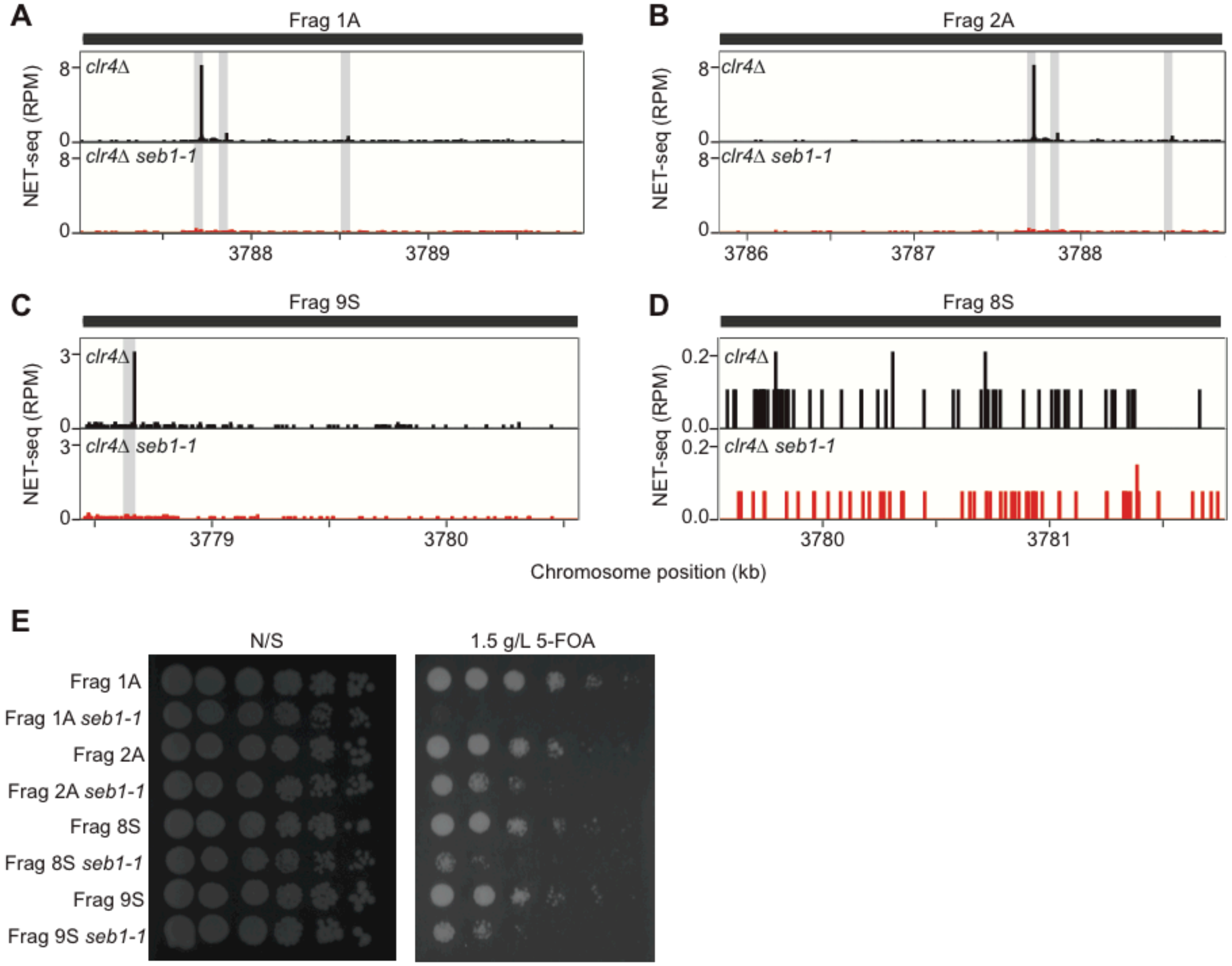

Supplemental Figure S5. Polymerase pauses and heterochromatin formation within centromere fragments are Seb1-dependent. A-D) NET-seq read density (RPM) for *clr4*Δ (black) and *clr4*Δ *seb1-1* (red) aligned to the four fragments in **Figure 2H** that display a capacity to induce heterochromatin formation in the context of the reporter construct shown in **Figure 2G**. **A)** *Frag1A*; **B)** *Frag2A*; **C)** *Frag9S*; **D)** *Frag8S*. Grey bars represent locations of NET-seq peaks/RNAPII pause sites present in the *clr4*Δ strain. **E)** 5-FOA spotting assay for Fragments with or without the mutant *seb1-1* allele.
