# Supplementary material for: Polymerase pausing induced by sequence-specific RNA-binding protein drives heterochromatin assembly
Source: Genes Dev. 2018 Jul 1;32(13-14):953–64. doi: 10.1101/gad.310136.117 (PMC6075038; doi:10.1101/gad.310136.117)
Supplement: Supplemental Material [file supp_32.13-14.953_Supplemental_Fig_S9.pdf]

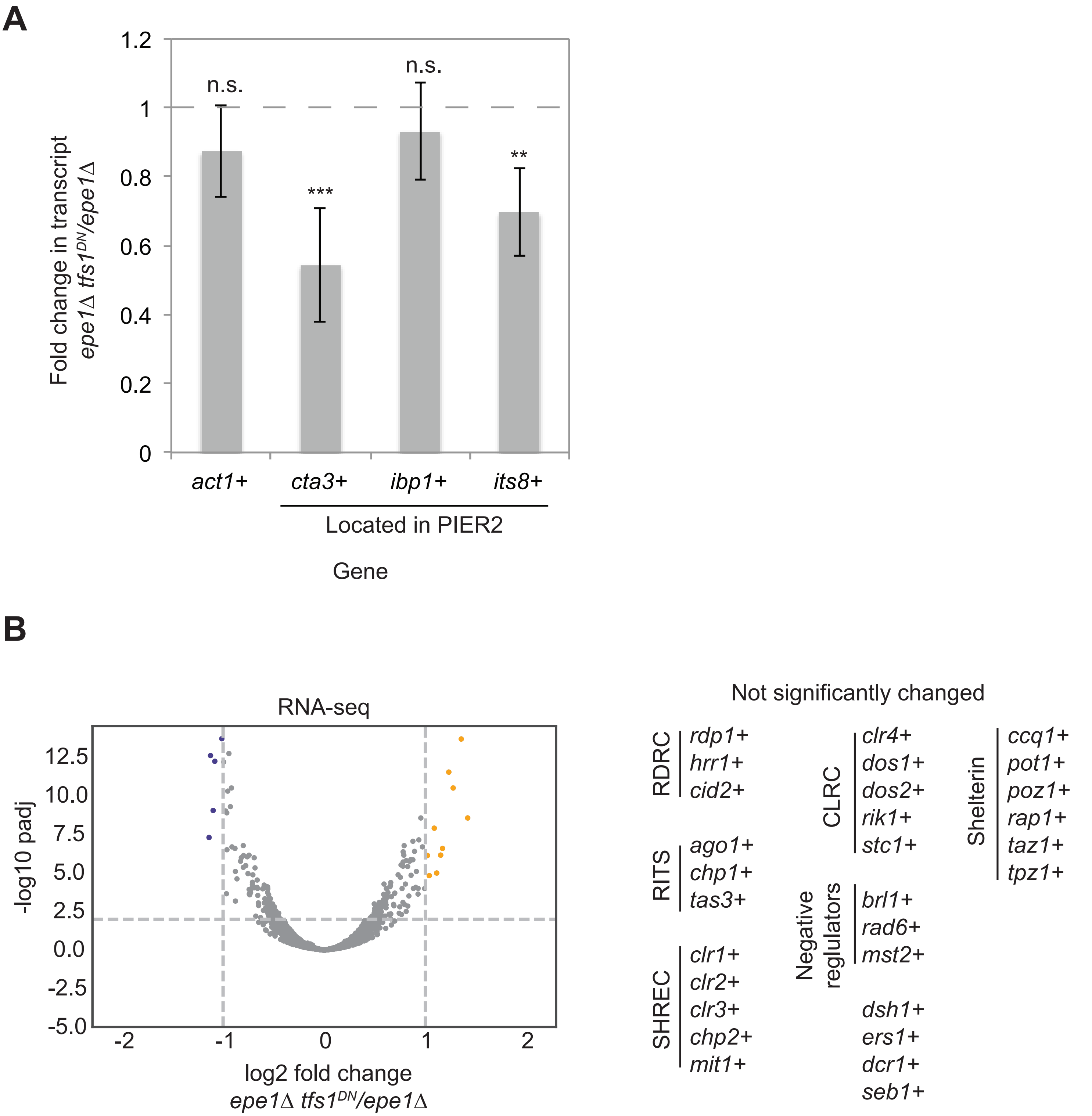

Supplemental Figure S9. TFIIS^{DN} expression silences genes within a PIER but does not alter expression of heterochromatin factors. A) Significantly

decreased transcript levels observed in two genes (*cta3*$^+$ and *its8*$^+$, but not *ibp1*$^+$) found within PIER2 (*act1*$^+$ is a control for no significant change). ***p<0.001, **p=0.0014. Dotted line = no change. **B)** RNA-seq volcano plot of log2 fold changes in transcript levels in *epe1*Δ *tfs1*^{DN} (Isolate 2 – containing PIER2) compared to *epe1*Δ. 11 genes have significantly lower expression in *epe1*Δ *tfs1*^{DN} (blue dots); 15 genes have significantly higher expression in *epe1*Δ *tfs1*^{DN} (orange dots). padj = adjusted p-value obtained from DESeq2. (right) No significant changes in transcript levels were observed in heterochromatin factors.
