# Supplementary material for: Polymerase pausing induced by sequence-specific RNA-binding protein drives heterochromatin assembly
Source: Genes Dev. 2018 Jul 1;32(13-14):953–64. doi: 10.1101/gad.310136.117 (PMC6075038; doi:10.1101/gad.310136.117)
Supplement: Supplemental Material [file supp_32.13-14.953_Supplemental_Table_S3.pdf]

Supplemental Table S3. List of chromosome coordinates of the fragments used in this study from the right arm of centromere I.

Fragment	Chromosome	Chromosome coordinate
Fragment 1	1	3787059..3789869
Fragment 2	1	3785843..3788836
Fragment 3	1	3784847..3787081
Fragment 4	1	3783812..3785861
Fragment 5	1	3782652..3784867
Fragment 6	1	3781725..3783833
Fragment 7	1	3780535..3782675
Fragment 8	1	3779557..3781747
Fragment 9	1	3778457..3780555
