# Supplementary material for: Polymerase pausing induced by sequence-specific RNA-binding protein drives heterochromatin assembly
Source: Genes Dev. 2018 Jul 1;32(13-14):953–64. doi: 10.1101/gad.310136.117 (PMC6075038; doi:10.1101/gad.310136.117)
Supplement: Supplemental Material [file supp_32.13-14.953_Supplemental_Fig_S3.pdf]

Supplemental Figure S3. *Parsa_Supplemental_FigS3*

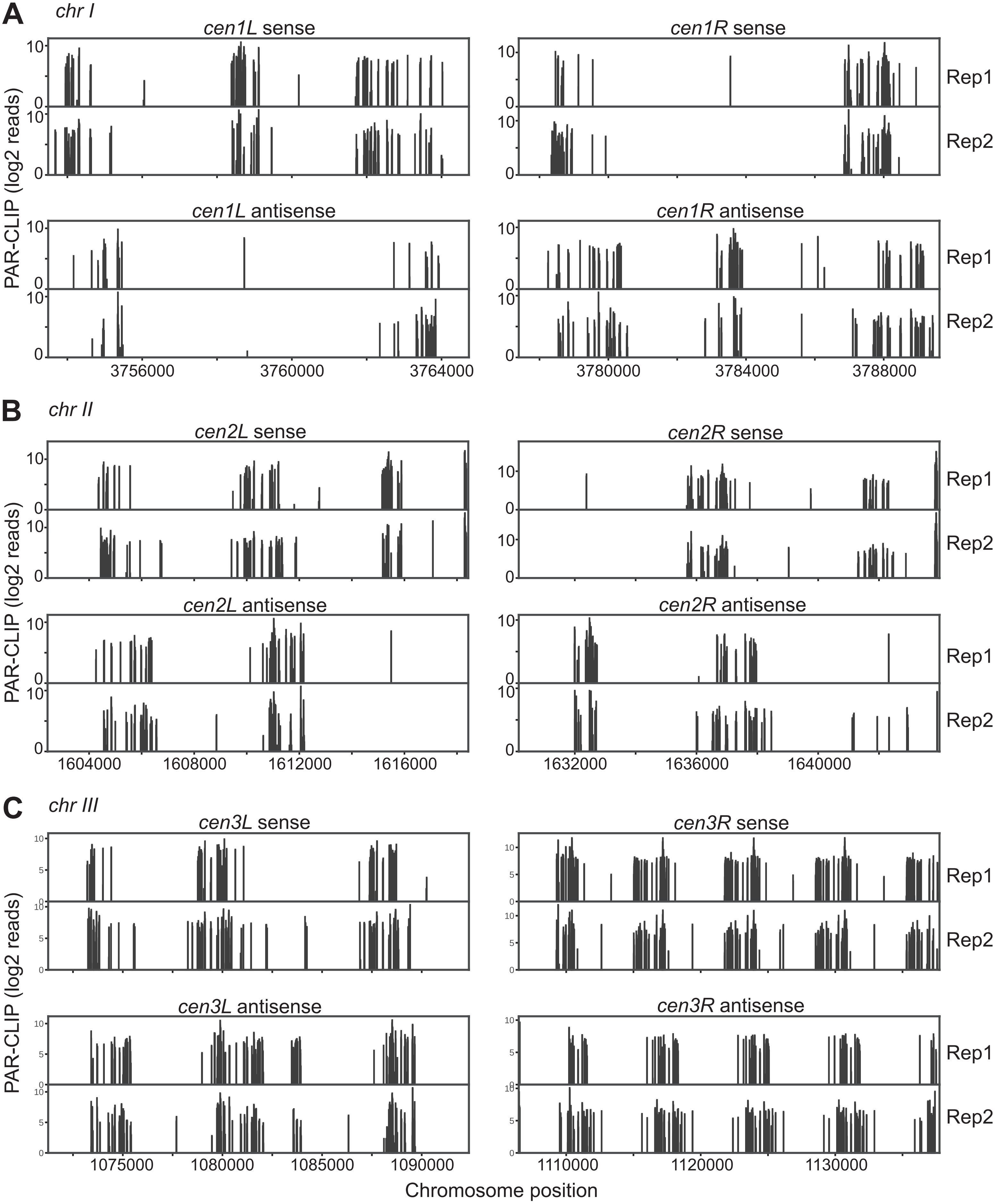

Supplemental Figure S3. PAR-CLIP analysis of Seb1 at all three centromeres. A-C) Seb1 PAR-CLIP clusters are present at centromeres 1 (**A**), 2 (**B**) and 3 (**C**). Centromere left (cenL) and right (cenR) arms are depicted for two replicates (Rep 1 and Rep 2) in both sense and antisense orientations.
