# Supplementary material for: Polymerase pausing induced by sequence-specific RNA-binding protein drives heterochromatin assembly
Source: Genes Dev. 2018 Jul 1;32(13-14):953–64. doi: 10.1101/gad.310136.117 (PMC6075038; doi:10.1101/gad.310136.117)
Supplement: Supplemental Material [file supp_32.13-14.953_Supplemental_Fig_S1.pdf]

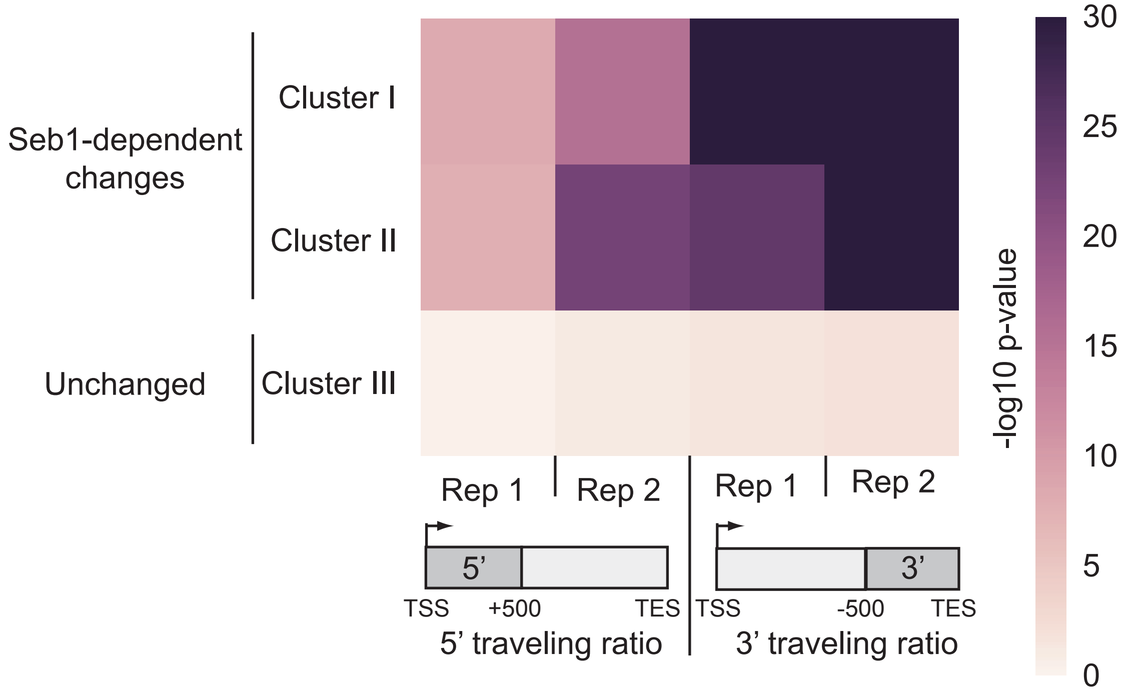

Supplemental Figure S1. *seb1-1* mutant displays statistically significant changes in 5' and 3' RNAPII traveling ratios. Heat map of –log10 p-values of KS tests comparing *clr4*Δ to *clr4*Δ *seb1-1* traveling ratios from each set of clusters in (**Figure 1D**). Clusters I and II display significant Seb1-dependent changes. Each replicate is represented (Rep 1 and Rep 2) for both 5' and 3' traveling ratios.
