# Supplementary material for: Polymerase pausing induced by sequence-specific RNA-binding protein drives heterochromatin assembly
Source: Genes Dev. 2018 Jul 1;32(13-14):953–64. doi: 10.1101/gad.310136.117 (PMC6075038; doi:10.1101/gad.310136.117)
Supplement: Supplemental Material [file supp_32.13-14.953_Supplemental_Fig_S8.pdf]

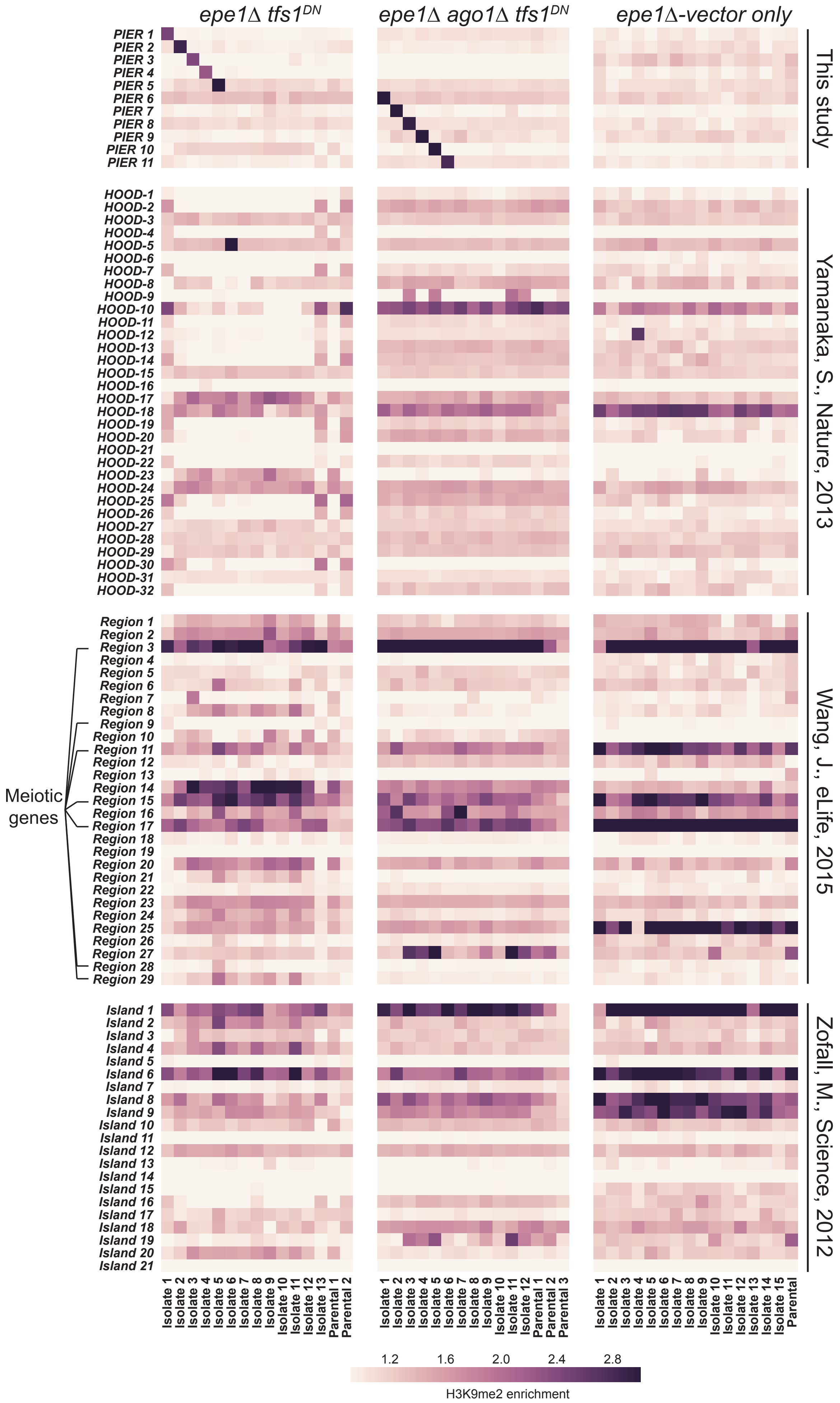
epe1Δ tfs1DN
epe1Δ ago1Δ tfs1DN
epe1Δ-vector only
PIER 1 – PIER 11
This study
HOOD-1 – HOOD-32
Yamanaka, S., Nature, 2013
Region 1 – Region 29
Meiotic genes
Wang, J., eLife, 2015
Island 1 – Island 21
Zofall, M., Science, 2012
Isolate 1 – Isolate 13, Parental 1, Parental 2
Isolate 1 – Isolate 12, Parental 1, Parental 2, Parental 3
Isolate 1 – Isolate 15, Parental
1.2 1.6 2.0 2.4 2.8
H3K9me2 enrichment

Supplemental Figure S8. H3K9me enrichment at known heterochromatin nucleation sites and PIERs. PIERs and known sites of heterochromatin nucleation were analyzed for H3K9me enrichment (see Methods). All isolates and parental strains for each genotype are depicted. HOODs, H3K9me islands, and meiotic genes are indicated.
