# Supplementary material for: Polymerase pausing induced by sequence-specific RNA-binding protein drives heterochromatin assembly
Source: Genes Dev. 2018 Jul 1;32(13-14):953–64. doi: 10.1101/gad.310136.117 (PMC6075038; doi:10.1101/gad.310136.117)
Supplement: Supplemental Material [file supp_32.13-14.953_Supplemental_Fig_S12.pdf]

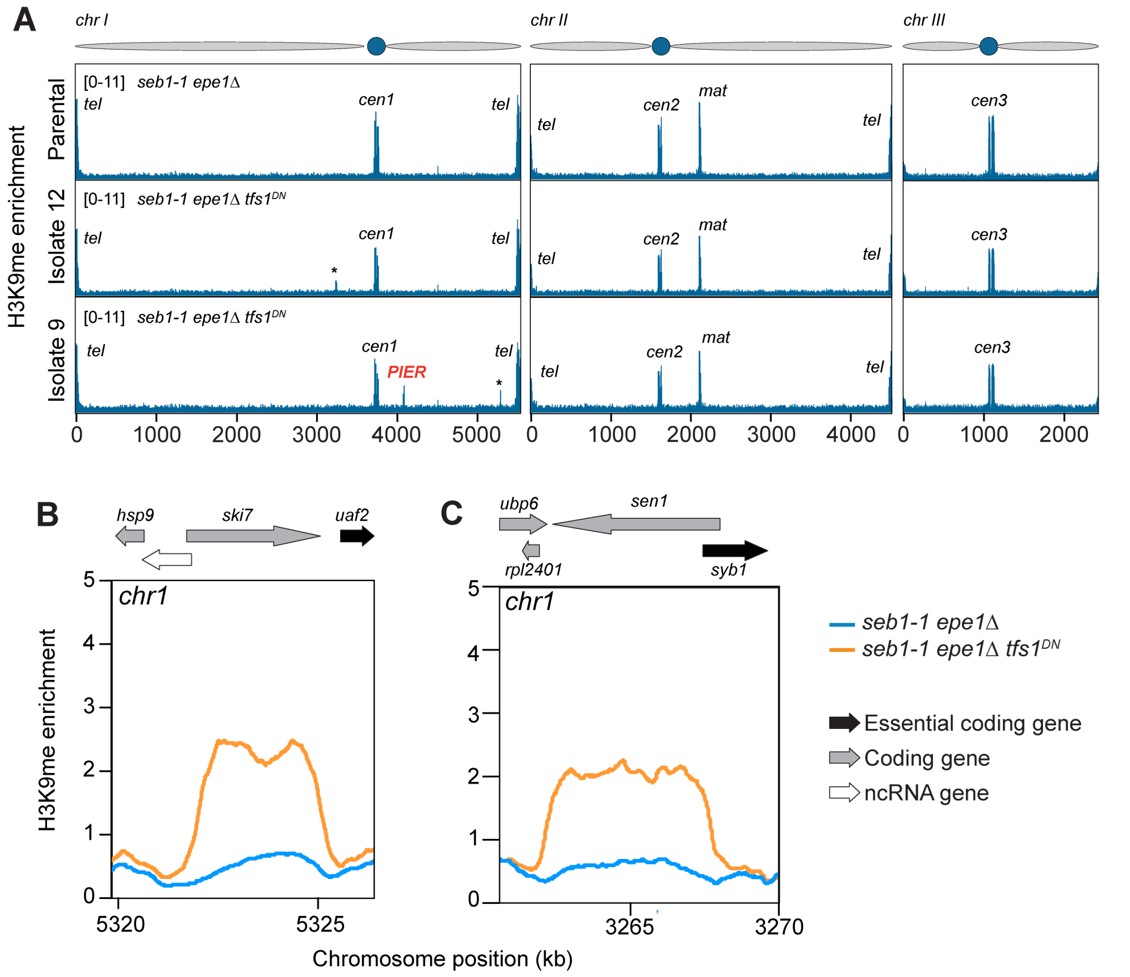


**Supplemental Figure S12. PIERs require Seb1 for assembly and enrichment of the H3K9me signal. A)** Genome-wide browser images of H3K9me enrichment. Asterisks demarcate regions of heterochromatin that do not pass our strict cutoffs for PIERs. **B** and **C**) Genome browser images of the two asterisks-marked loci in (A). H3K9me enrichment for *seb1-1 epe1*Δ (blue) and *seb1-1 epe1*Δ *tfs1*$^{DN}$ (orange).
