# Supplementary material for: Polymerase pausing induced by sequence-specific RNA-binding protein drives heterochromatin assembly
Source: Genes Dev. 2018 Jul 1;32(13-14):953–64. doi: 10.1101/gad.310136.117 (PMC6075038; doi:10.1101/gad.310136.117)
Supplement: Supplemental Material [file supp_32.13-14.953_Supplemental_Fig_S6.pdf]

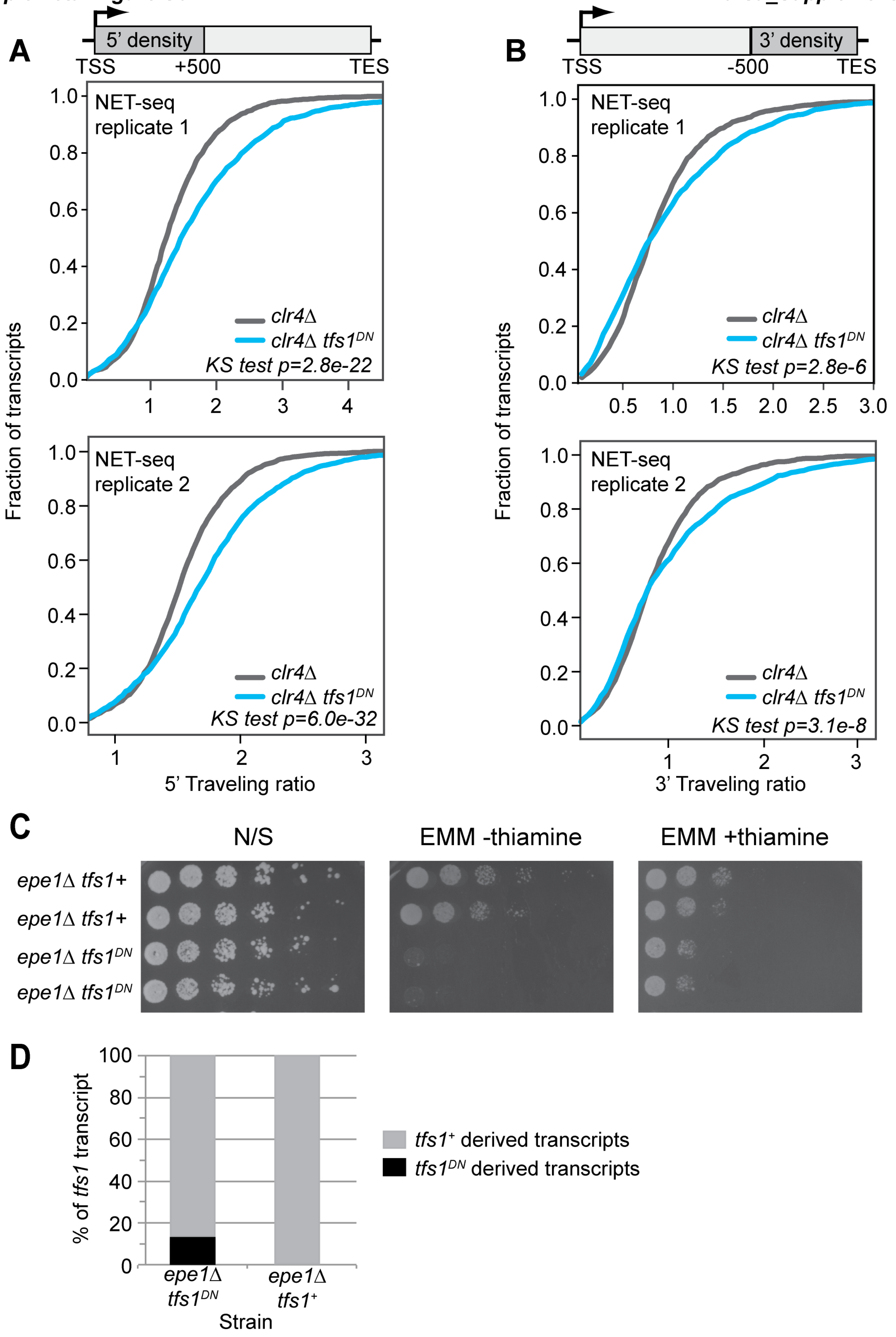
A
5' density
TSS
+500
TES
NET-seq replicate 1
clr4Δ
clr4Δ tfs1DN
KS test p=2.8e-22
NET-seq replicate 2
clr4Δ
clr4Δ tfs1DN
KS test p=6.0e-32
Fraction of transcripts
5' Traveling ratio
B
3' density
TSS
-500
TES
NET-seq replicate 1
clr4Δ
clr4Δ tfs1DN
KS test p=2.8e-6
NET-seq replicate 2
clr4Δ
clr4Δ tfs1DN
KS test p=3.1e-8
Fraction of transcripts
3' Traveling ratio
C
N/S
EMM -thiamine
EMM +thiamine
epe1Δ tfs1+
epe1Δ tfs1+
epe1Δ tfs1DN
epe1Δ tfs1DN
D
% of tfs1 transcript
epe1Δ tfs1DN
epe1Δ tfs1+
Strain
tfs1+ derived transcripts
tfs1DN derived transcripts

Supplemental Figure S6. ***pnmt1^{+}-tfs1^{DN}*** **expression impacts traveling ratios genome wide. A)** Cumulative distribution function (cdf) plots of 5' traveling ratios for *clr4*Δ (dark grey) to *clr4*Δ *tfs1^{DN}* (blue) NET-seq strains for two replicates. KS tests were conducted for p-values. **B)** Cumulative distribution function (cdf) plots of 3' traveling ratios for *clr4*Δ (dark grey) to *clr4*Δ *tfs1^{DN}* (blue) NET-seq strains for two replicates. KS tests were conducted for p-values. **C)** Growth assays on *epe1*Δ *tfs1^{+}* and *epe1*Δ *tfs1^{DN}* strains. Cells were plated on non-selective rich YS medium (N/S), EMM medium in the absence of thiamine (EMM -thiamine), and EMM medium containing 15uM thiamine (EMM +thiamine). **D)** Percent of total *tfs1* reads derived from the *tfs1^{+}* (grey) and *tfs1^{DN}* alleles (black) in *pnmt1*+ repressible conditions (EMM 15uM thiamine) obtained from RNA-seq analysis of *epe1*Δ *tfs1^{DN}* and *epe1*Δ *tfs1^{+}* strains (no reads corresponding to the *tfs1^{DN}* allele were observed in the *epe1*Δ *tfs1^{+}* strain).
