# Supplementary material for: Polymerase pausing induced by sequence-specific RNA-binding protein drives heterochromatin assembly
Source: Genes Dev. 2018 Jul 1;32(13-14):953–64. doi: 10.1101/gad.310136.117 (PMC6075038; doi:10.1101/gad.310136.117)
Supplement: Supplemental Material [file supp_32.13-14.953_Supplemental_Fig_S10.pdf]

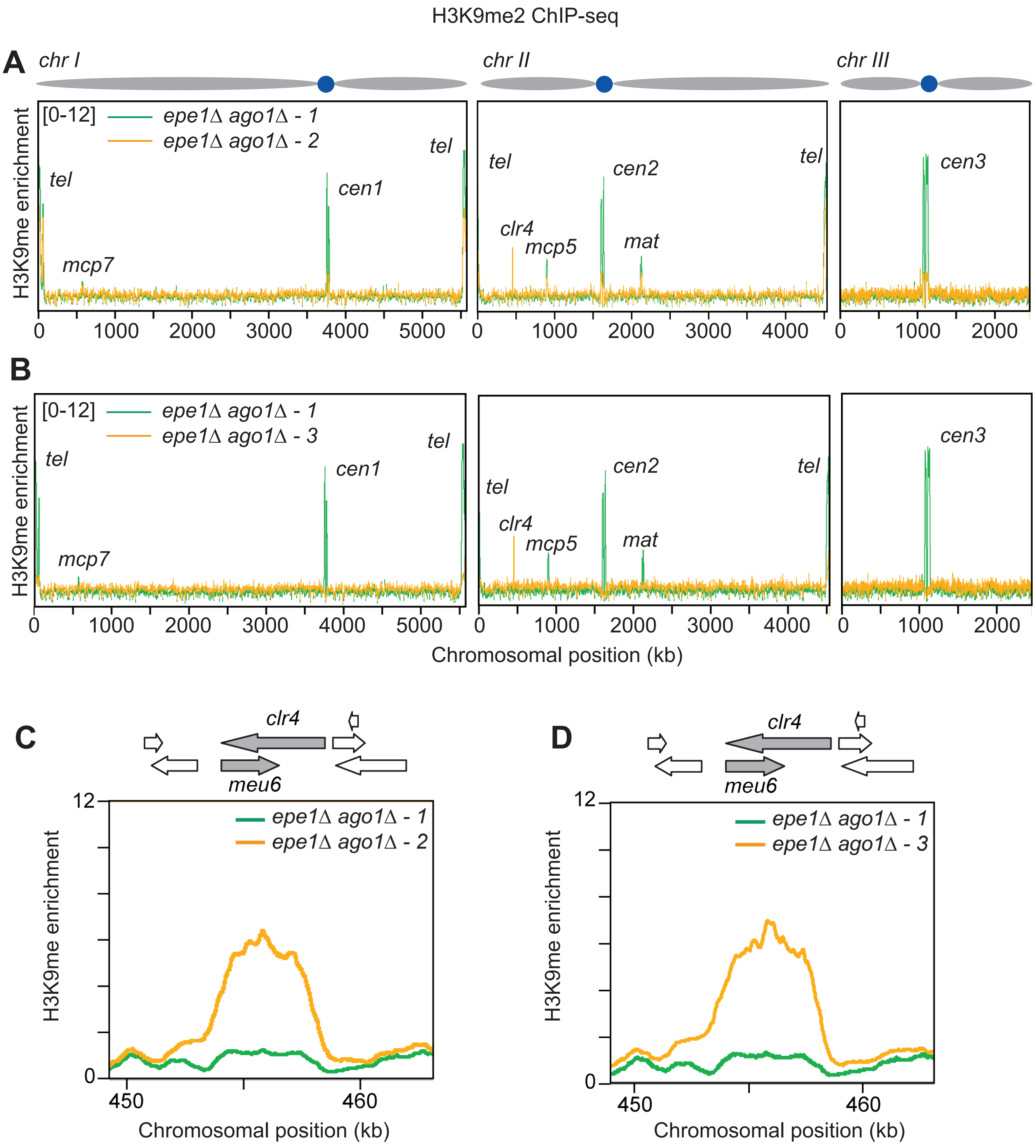
H3K9me2 ChIP-seq
A
chr I
chr II
chr III
H3K9me enrichment
[0-12]
epe1Δ ago1Δ - 1
epe1Δ ago1Δ - 2
tel
mcp7
cen1
tel
clr4
mcp5
cen2
mat
cen3
0 1000 2000 3000 4000 5000
B
epe1Δ ago1Δ - 3
Chromosomal position (kb)
C
clr4
meu6
12
0
450
460
D

Supplemental Figure S10. *epe1Δ ago1Δ* strains can adaptively silence the *clr4*$^{+}$ locus. A-B) Whole genome H3K9me2 enrichment plots for two independent *epe1Δ ago1Δ* strains that accumulated H3K9me2 over the *clr4*$^{+}$ locus, *epe1Δ ago1Δ-2* (**A**) and *epe1Δ ago1Δ-3* (**B**) (in orange), compared to a isolate with normal distribution of H3K9me2 across the genome, *epe1Δ ago1Δ-1* (in green). **C-D)** Genome browser image of the enrichment of H3K9me2 over *clr4*$^{+}$ locus in *epe1Δ ago1Δ-2* (**C**) and *epe1Δ ago1Δ-3* (**D**).
