# Supplementary material for: Polymerase pausing induced by sequence-specific RNA-binding protein drives heterochromatin assembly
Source: Genes Dev. 2018 Jul 1;32(13-14):953–64. doi: 10.1101/gad.310136.117 (PMC6075038; doi:10.1101/gad.310136.117)
Supplement: Supplemental Material [file supp_32.13-14.953_Supplemental_Table_S6.pdf]

Supplemental Table S6. Known heterochromatin recruitment sites

Chromosome	Start	End	Name	Reference
I	64559	66980	Region 1	Wang, J. eLife, 2015
I	241008	247574	Region 2	Wang, J. eLife, 2015
I	578392	581553	Region 3	Wang, J. eLife, 2015
I	1024002	1029993	Region 4	Wang, J. eLife, 2015
I	1892505	1896752	Region 5	Wang, J. eLife, 2015
I	2442000	2452000	Region 6	Wang, J. eLife, 2015
I	2517000	2527000	Region 7	Wang, J. eLife, 2015
I	3646000	3650000	Region 8	Wang, J. eLife, 2015
I	3726000	3730000	Region 9	Wang, J. eLife, 2015
I	4529389	4531031	Region 10	Wang, J. eLife, 2015
I	4530000	4540000	Region 11	Wang, J. eLife, 2015
I	4642290	4647689	Region 12	Wang, J. eLife, 2015
I	4648000	4656000	Region 13	Wang, J. eLife, 2015
II	350692	352369	Region 14	Wang, J. eLife, 2015
II	892000	904000	Region 15	Wang, J. eLife, 2015
II	1309072	1311834	Region 16	Wang, J. eLife, 2015
II	1473125	1475285	Region 17	Wang, J. eLife, 2015
II	1863000	1871000	Region 18	Wang, J. eLife, 2015
II	2196424	2202652	Region 19	Wang, J. eLife, 2015
II	2335176	2337787	Region 20	Wang, J. eLife, 2015
II	3622580	3628658	Region 21	Wang, J. eLife, 2015
II	3668000	3678000	Region 22	Wang, J. eLife, 2015
II	3682000	3688000	Region 23	Wang, J. eLife, 2015
II	4252276	4255310	Region 24	Wang, J. eLife, 2015
III	275261	277340	Region 25	Wang, J. eLife, 2015
III	386020	390514	Region 26	Wang, J. eLife, 2015
III	1034000	1042000	Region 27	Wang, J. eLife, 2015
III	1451701	1457855	Region 28	Wang, J. eLife, 2015
III	2364000	2372000	Region 29	Wang, J. eLife, 2015
I	578000	582000	*Island 1*	Zofall, M. Science, 2012
I	2447000	2449000	*Island 2*	Zofall, M. Science, 2012
I	2521000	2525000	*Island 3*	Zofall, M. Science, 2012
I	3647000	3651000	*Island 4*	Zofall, M. Science, 2012
I	3727000	3730000	*Island 5*	Zofall, M. Science, 2012
I	4534000	4540000	*Island 6*	Zofall, M. Science, 2012
I	4653000	4656000	*Island 7*	Zofall, M. Science, 2012
II	898000	903000	*Island 8*	Zofall, M. Science, 2012
II	1472000	1479000	*Island 9*	Zofall, M. Science, 2012
II	1551000	1554000	*Island 10*	Zofall, M. Science, 2012
II	1670000	1680000	*Island 11*	Zofall, M. Science, 2012
II	1692000	1698000	*Island 12*	Zofall, M. Science, 2012
II	1869000	1873000	*Island 13*	Zofall, M. Science, 2012
II	2199000	2202000	*Island 14*	Zofall, M. Science, 2012
II	2338000	2342000	*Island 15*	Zofall, M. Science, 2012
II	3628000	3631000	*Island 16*	Zofall, M. Science, 2012
II	3640000	3642000	*Island 17*	Zofall, M. Science, 2012
III	958000	968000	*Island 18*	Zofall, M. Science, 2012

III	1036000	1040000	*Island 19*	Zofall, M. Science, 2012
III	2369000	2371000	*Island 20*	Zofall, M. Science, 2012
III	2422000	2424000	*Island 21*	Zofall, M. Science, 2012
I	1465847	1469848	*HOOD-1*	Yamanaka, S. Nature, 2013
I	1564163	1568414	*HOOD-2*	Yamanaka, S. Nature, 2013
I	2544835	2561773	*HOOD-3*	Yamanaka, S. Nature, 2013
I	2927156	2941954	*HOOD-4*	Yamanaka, S. Nature, 2013
I	2977013	2988899	*HOOD-5*	Yamanaka, S. Nature, 2013
I	2994807	3009469	*HOOD-6*	Yamanaka, S. Nature, 2013
I	3361499	3365727	*HOOD-7*	Yamanaka, S. Nature, 2013
I	3736902	3743575	*HOOD-8*	Yamanaka, S. Nature, 2013
I	3791825	3796114	*HOOD-9*	Yamanaka, S. Nature, 2013
I	3996353	4000615	*HOOD-10*	Yamanaka, S. Nature, 2013
I	4022326	4026545	*HOOD-11*	Yamanaka, S. Nature, 2013
I	5069824	5083205	*HOOD-12*	Yamanaka, S. Nature, 2013
I	5191103	5195325	*HOOD-13*	Yamanaka, S. Nature, 2013
I	5195656	5199909	*HOOD-14*	Yamanaka, S. Nature, 2013
I	5234000	5250176	*HOOD-15*	Yamanaka, S. Nature, 2013
II	91600	101684	*HOOD-16*	Yamanaka, S. Nature, 2013
II	347505	354250	*HOOD-17*	Yamanaka, S. Nature, 2013
II	898107	902233	*HOOD-18*	Yamanaka, S. Nature, 2013
II	1812684	1816937	*HOOD-19*	Yamanaka, S. Nature, 2013
II	1965175	1969519	*HOOD-20*	Yamanaka, S. Nature, 2013
II	2126590	2128479	*HOOD-21*	Yamanaka, S. Nature, 2013
II	4414469	4418768	*HOOD-22*	Yamanaka, S. Nature, 2013
II	4442538	4449562	*HOOD-23*	Yamanaka, S. Nature, 2013
III	173841	176400	*HOOD-24*	Yamanaka, S. Nature, 2013
III	254411	256353	*HOOD-25*	Yamanaka, S. Nature, 2013
III	778123	782331	*HOOD-26*	Yamanaka, S. Nature, 2013
III	1047657	1056145	*HOOD-27*	Yamanaka, S. Nature, 2013
III	1168500	1176000	*HOOD-28*	Yamanaka, S. Nature, 2013
III	1179500	1182650	*HOOD-29*	Yamanaka, S. Nature, 2013
III	1196050	1196500	*HOOD-30*	Yamanaka, S. Nature, 2013
III	1763512	1775613	*HOOD-31*	Yamanaka, S. Nature, 2013
III	2320230	2324503	*HOOD-32*	Yamanaka, S. Nature, 2013
