# Supplementary material for: Polymerase pausing induced by sequence-specific RNA-binding protein drives heterochromatin assembly
Source: Genes Dev. 2018 Jul 1;32(13-14):953–64. doi: 10.1101/gad.310136.117 (PMC6075038; doi:10.1101/gad.310136.117)
Supplement: Supplemental Material [file supp_32.13-14.953_Supplemental_Fig_S4.pdf]

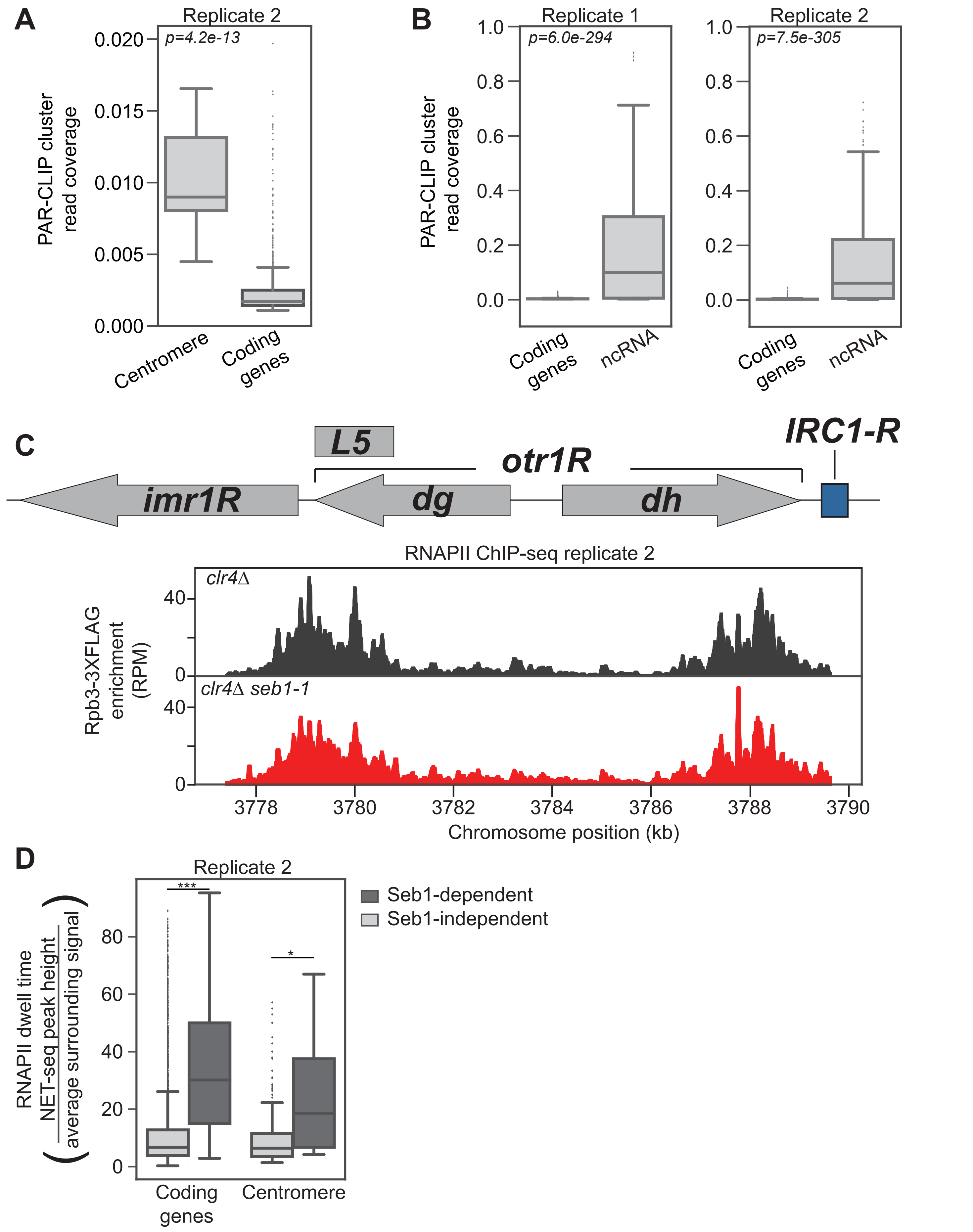
A
Replicate 2
p=4.2e-13
0.020
0.015
0.010
0.005
0.000
PAR-CLIP cluster read coverage
Centromere
Coding genes
B
Replicate 1
p=6.0e-294
Replicate 2
p=7.5e-305
1.0
0.8
0.6
0.4
0.2
0.0
PAR-CLIP cluster read coverage
Coding genes
ncRNA
C
L5
otr1R
IRC1-R
imr1R
dg
dh
RNAPII ChIP-seq replicate 2
clr4Δ
clr4Δ seb1-1
Rpb3-3XFLAG enrichment (RPM)
40
0
3778
3780
3782
3784
3786
3788
3790
Chromosome position (kb)
D
Replicate 2
Seb1-dependent
Seb1-independent
RNAPII dwell time
NET-seq peak height / average surrounding signal
80
60
40
20
0
***
*
Coding genes
Centromere

Supplemental Figure S4. Seb1 induces RNAPII pauses with long dwell times throughout the genome. A) Seb1 PAR-CLIP cluster read coverage at the centromeres compared to coding genes for the second replicate. **B)** Seb1 PAR-CLIP cluster read coverage at ncRNAs (including sn/snoRNAs, tRNAs) compared to coding genes. **C)** Replicate 2 data for RNAPII levels based on ChIP-seq of Rpb3-3xFLAG with reads aligned to the right arm of centromere 1. Comparing RNAPII enrichment in *clr4*Δ (black) and *clr4*Δ *seb1-1* (red). **D)** RNAPII dwell time analysis for centromeres and coding genes in a second replicate (*p=0.026; ***p=1.8e-56).
