# Supplementary material for: Polymerase pausing induced by sequence-specific RNA-binding protein drives heterochromatin assembly
Source: Genes Dev. 2018 Jul 1;32(13-14):953–64. doi: 10.1101/gad.310136.117 (PMC6075038; doi:10.1101/gad.310136.117)
Supplement: Supplemental Material [file supp_32.13-14.953_Supplemental_Fig_S2.pdf]

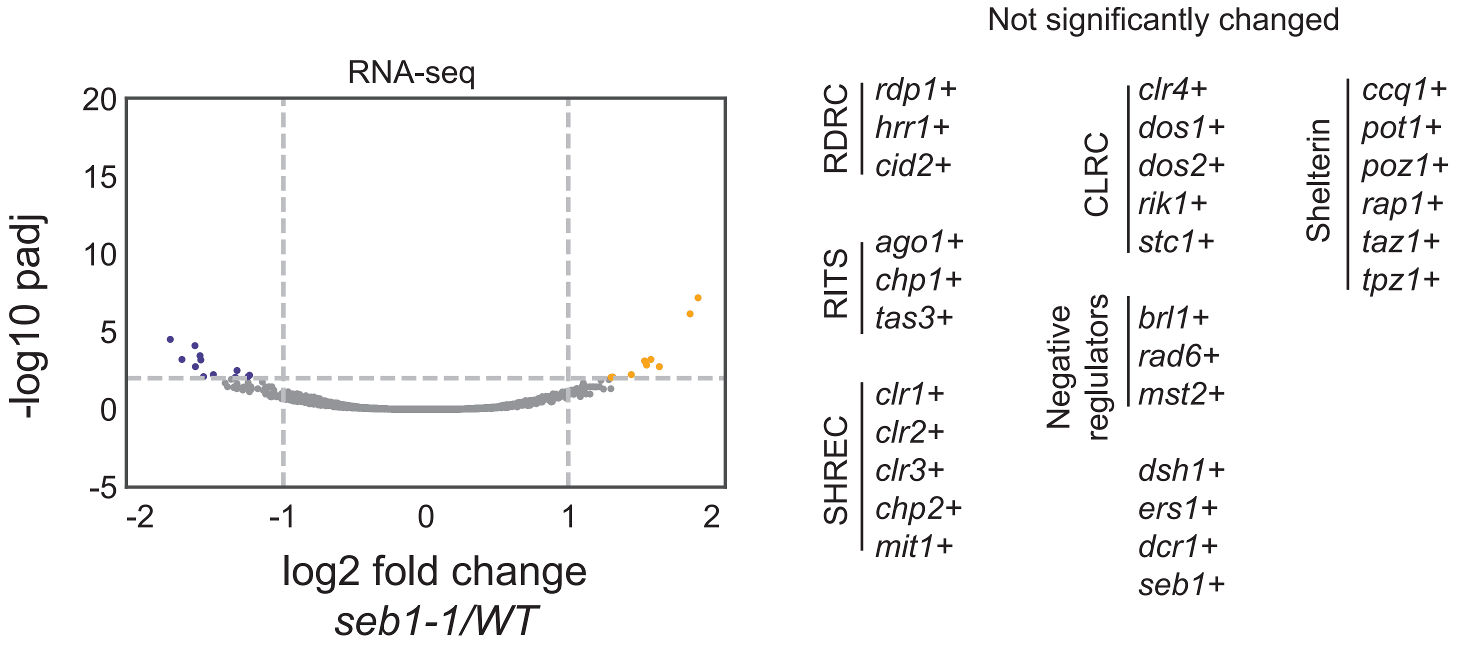

Supplemental Figure S2. RNA-seq anlaysis of *seb1-1*. (left) RNA-seq volcano plots of log2 fold changes in transcript levels in *seb1-1* compared to WT. 15 genes have significantly lower expression in *seb1-1* (blue dots); 9 genes have significantly higher expression in *seb1-1* (orange dots). padj = adjusted p-value obtained from DESeq2. (right) No significant changes in transcript levels were observed in factors known to play a role in heterochromatin formation.
