# Supplementary material for: Polymerase pausing induced by sequence-specific RNA-binding protein drives heterochromatin assembly
Source: Genes Dev. 2018 Jul 1;32(13-14):953–64. doi: 10.1101/gad.310136.117 (PMC6075038; doi:10.1101/gad.310136.117)
Supplement: Supplemental Material [file supp_32.13-14.953_Supplemental_Fig_S13.pdf]

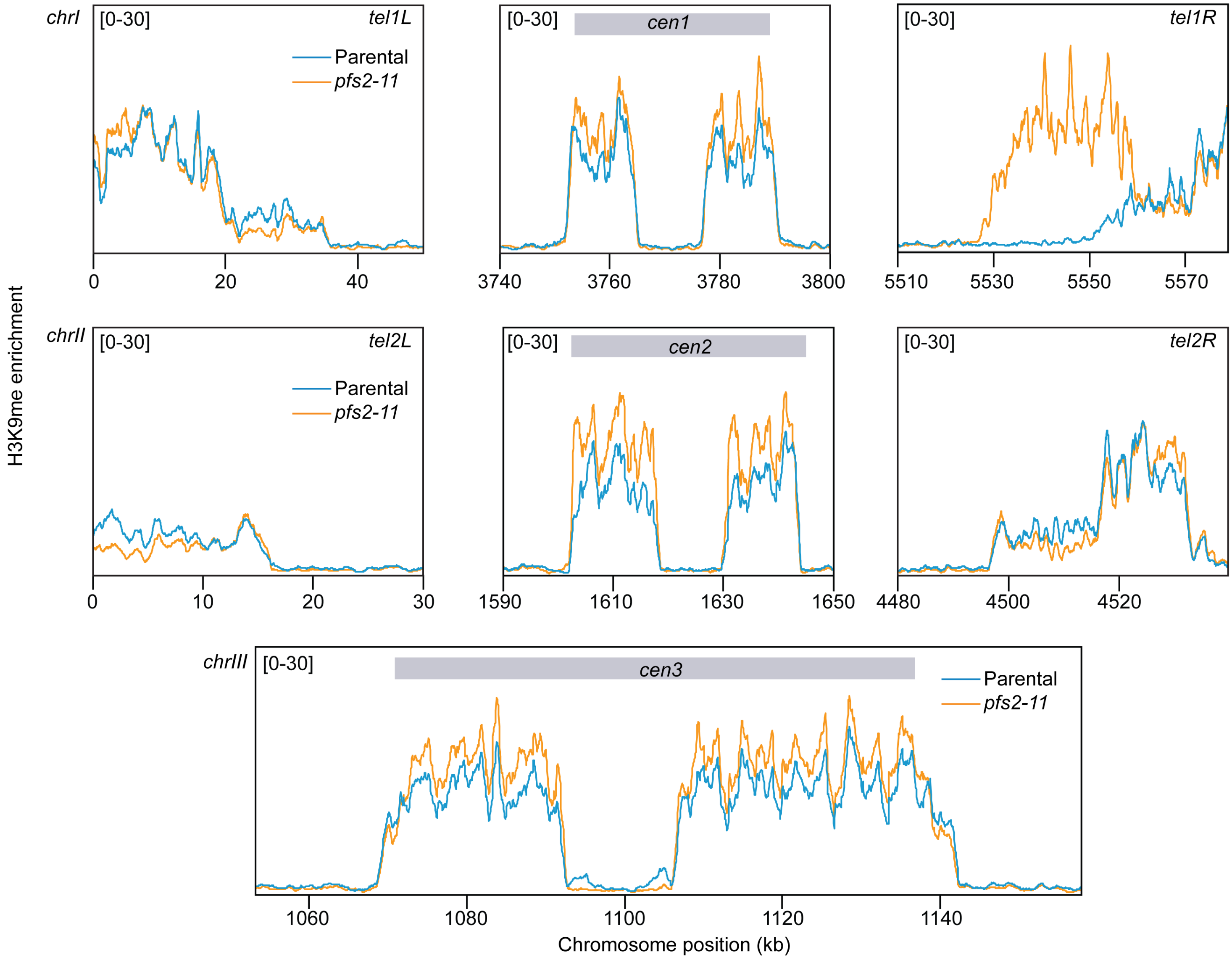

Supplemental Figure S13. Mutations in CPA machinery result in increased H3K9me enrichment at constitutive heterochromatic loci. Replicate data for H3K9me ChIP-seq for *pfs2-11* (Figure 5B-D). Telomeres from chromosomes I and II and centromeres from chromosomes I, II and II depicted. Parent (blue), *pfs2-11* mutant (orange).
